# Cortical processing modulation in DOC by preferred music-coupled 40 Hz binaural stimulation: an exploratory EEG-fNIRS study

**DOI:** 10.3389/fpsyg.2026.1783416

**Published:** 2026-07-13

**Authors:** Jie Wang, Sheng Qu, Yancai Zhou, Yanan Wang, Laigang Huang, Fanshuo Zeng

**Affiliations:** 1 Department of Rehabilitation, The Second Qilu Hospital of Shandong University, Jinan, Shandong, China; 2 Department of Rehabilitation, Heze Third People’s Hospital, Heze, Shandong, China; 3 Baotuquan Campus, Shandong University, Jinan, Shandong, China

**Keywords:** binaural beat therapy, disorders of consciousness, electroencephalogram, functional near-infrared spectroscopy, machine learning, music therapy

## Abstract

**Objective:**

To determine if 40 Hz binaural beat therapy (BBT) combined with preferred music enhances neural complexity and connectivity in disorders of consciousness (DOC).

**Methods:**

In a randomized controlled trial, 40 patients with DOC, including minimally conscious state (MCS) and vegetative state/unresponsive wakefulness syndrome (VS/UWS), received either daily 40 Hz BBT with preferred music (*n* = 21) or standard rehabilitation (*n* = 19). Primary outcomes included Coma Recovery Scale-Revised (CRS-R) scores, electroencephalography (EEG)-based approximate entropy (ApEn), cross-ApEn (C-ApEn), and functional near-infrared spectroscopy (fNIRS)-based average functional connectivity (FC) of functional connectivity (HbO).

**Results:**

Both groups showed CRS-R improvements (*p* < 0.05). The 40 Hz BBT group demonstrated significant enhancement in prefrontal pole of ApEn (*p* < 0.05, Cohen’s *d* = 0.65), central-prefrontal pole of C-ApEn (*p* < 0.05, Cohen’s *d* = 0.56), and average FC of HbO (*p* < 0.05, Cohen’s *d* = 0.86), with moderate to large effect sizes. In contrast, the control group showed smaller and non-significant effect sizes for these measures (Cohen’s *d* = 0.27–0.40). These effects were absent in VS/UWS patients of the 40 Hz BBT music therapy group. The prefrontal pole of ApEn correlated strongest with clinical outcomes (*R* = 0.342, *p* = 0.031).

**Conclusion:**

The findings suggest that 40 Hz BBT combined with preferred music may enhance neural complexity and functional connectivity in patients with DOC, though these effects were not observed in VS/UWS patients receiving the 40 Hz BBT music therapy.

**Clinical trial registration:**

https://www.chictr.org.cn/, identifier ChiCTR2300079310.

## 1 Introduction

Disorders of consciousness (DOC), arising from severe brain injuries such as trauma, stroke, or hypoxia, are characterized by disruptions in wakefulness and awareness. Clinically, patients are stratified along a recovery spectrum ranging from coma to vegetative state/unresponsive wakefulness syndrome (VS/UWS) and minimally conscious state (MCS) (Zhao, 2018). Prolonged DOC often leads to environmental deprivation, a state of reduced sensory and social input due to medical isolation and neurological impairment. This deprivation may exacerbate neural dysfunction and delay recovery (Oh and Seo, 2003). Therapeutic approaches based on enriched environments counteract this deprivation through structured sensory input (Megha et al., 2013). Targeted sensory stimulation (e.g., auditory and tactile) accelerates recovery, as evidenced by faster transitions to higher consciousness states and improved functional outcomes mediated by enhanced neuroplasticity mechanisms (Oh and Seo, 2003; Sasaki et al., 2022).

Notably, auditory stimuli have garnered particular attention because of their persistence as the last sensory modality to diminish in progressive brain dysfunction, a phenomenon attributed to the resilience of subcortical auditory pathways and documented in electrophysiological studies of DOC (Çevik and Namik, 2018; Blundon et al., 2020). Beyond generic auditory stimuli, music orchestrates cortico-subcortical neuromodulation through two mechanisms: (1) co-activation of auditory-limbic networks (primary auditory cortex/amygdala-hippocampal complex), and (2) dopaminergic-cholinergic neurotransmitter release in mesocortical pathways, which are neurochemical correlates of the emotional-arousal consciousness model (Rollnik and Altenmüller, 2014; Grimm and Kreutz, 2018). Crucially, autobiographical music (i.e., preferred music with strong personal mnemonics) potentiates cortical excitability by engaging episodic memory circuits, whereas generic preferred music primarily modulates limbic reward pathways (Qu et al., 2024). This neurobiological mechanism forms the foundational framework for our intervention in the DOC.

Binaural beat therapy (BBT) induces frequency-specific neuromodulation through interaural frequency disparity (Δf), generating phase-locked frequency-following responses in the medial superior olive, a brainstem-thalamic entrainment mechanism mediated by glutamatergic reticular projections (Jang and Choi, 2022; Lin et al., 2024; Engelbregt et al., 2021). Crucially, 40 Hz gamma-band BBT drives thalamocortical phase-amplitude coupling, facilitating large-scale synchronization, which is critical for global workspace integration and conscious processing (Kim et al., 2023; Wu et al., 2024). The functional relevance of this frequency is further supported by clinical evidence: the strength of gamma-band (~40 Hz) oscillatory activity in the cerebral cortex has been positively correlated with the level of consciousness in DOC patients, while also being linked to lucid dreaming in healthy individuals (Wu et al., 2022; Górska and Binder, 2019). Building on this framework, we designed a dual-pathway intervention combining top-down preferred music (engaging default mode network hubs) with bottom-up 40 Hz BBT (synchronizing thalamoreticular circuits), operationalizing predictive coding principles to restore consciousness through amplified gamma coherence and cortico-limbic resonance. This paradigm bridges oscillation-targeted neuromodulation with personalized sensory enrichment, offering a mechanistically grounded strategy for the rehabilitation of disorders of consciousness.

Accurate assessment of intervention-induced neural changes is essential for validating the efficacy of therapeutic approaches in patients with DOC. However, behavioral assessments alone are often insufficient, where cognitive-motor dissociation leads to misdiagnosis in 15% of patients with VS/UWS (Kondziella et al., 2016). Functional near-infrared spectroscopy (fNIRS), which monitors changes in cerebral oxygenation levels and hemodynamic responses, operates on the principle of tight coupling between neural activation and vascular processes (Rupawala et al., 2018). However, its temporal fidelity is constrained by hemodynamic delays (2–5 s latency) (Rupawala et al., 2018; Shu et al., 2023). Conversely, electroencephalography (EEG) achieves millisecond-scale temporal resolution (<10 ms), capturing metastable microstate transitions (Li et al., 2022). The integration of fNIRS and EEG therefore enables a multidimensional characterization of treatment-related cortical modulation, allowing for more robust evaluation of intervention outcomes in DOC patients (Su et al., 2023).

The main objective of this research was to explore the effect of 40 Hz-BBT music therapy on DOC patients and to assess the changes in the level of consciousness before and after the intervention using EEG, fNIRS, and Coma Recovery Scale-Revised (CRS-R) scores. By analyzing brain networks using fNIRS and exploring brain dynamics using EEG, we aim to shed light on the intricate interplay between music, brain function, and the state of consciousness. As an exploratory objective, machine learning algorithms will be used to integrate spatiotemporal features from fNIRS and EEG to develop predictive models for covert consciousness detection.

## 2 Materials and methods

### 2.1 Participants

This single-blind randomized controlled trial (RCT) enrolled 42 consecutive patients from the Rehabilitation Department of the Second Qilu Hospital of Shandong University between May 2024 and February 2025. The inclusion criteria were as follows: (1) diagnosis of VS/UWS or MCS confirmed by CRS-R; (2) age 18–85 years; (3) post-injury duration of 28 days to 18 months; (4) at least one CRS-R auditory subscale score; and (5) right-handedness. The exclusion criteria were as follows: (1) pre-injury hearing impairment, hemodynamic instability during monitoring, severe cerebral atrophy/hydrocephalus on imaging, (2) locked-in syndrome, (3) significant spasticity interfering with recordings, (4) pre-existing neuropsychiatric disorders, or (5) unverified cranial integrity. The study protocol was approved by the Institutional Review Board of the Second Qilu Hospital of Shandong University (Approval No. KYLL-2023-414) and registered in the Chinese Clinical Trial Registry (ChiCTR2300079310). All procedures were performed in accordance with the ethical principles of the Declaration of Helsinki. Informed consent was obtained from all subjects’ guardians prior to enrollment. Written informed consent was obtained from the family members of the patients with DOC.

Demographics, including age, sex, time since injury, lesion laterality, Coma Recovery Scale-Revised (CRS-R) scores, and cause of injury, were systematically recorded. For the CRS-R assessment, which evaluates six functional domains (auditory, visual, motor, verbal, communication, and arousal) through 23 hierarchically scored items (total score range: 0–23), a rigorous protocol was implemented: First, ≥2 trained clinicians conducted ≥5 repeated evaluations (1–2 daily over 1 week) (Wang et al., 2020). Subsequently, the highest single score was used to establish the diagnosis, whereas the mean scores from all assessments were utilized for statistical analysis.

### 2.2 Study design

**Figure 1:**
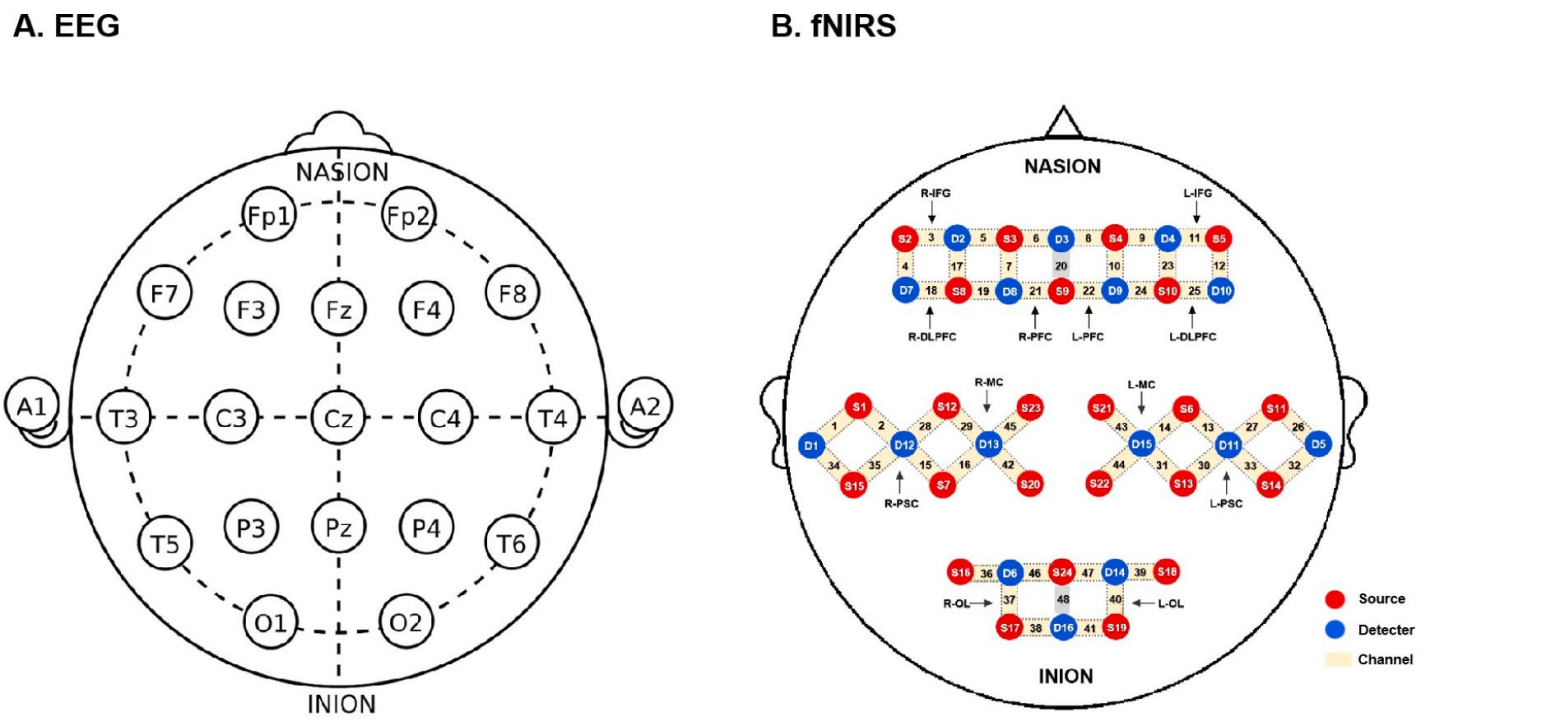
EEG electrode placement and fNIRS channel configuration. **(A)** EEG 10–20 system electrodes; **(B)** fNIRS channels with NASION landmarks. EEG, electroencephalography; fNIRS, functional near-infrared spectroscopy; L-DLPFC/R-DLPFC, the left/right dorsolateral prefrontal cortex; L-PFC/R-PFC, the left/right prefrontal cortex; L-IFG/R-IFG, the left/right inferior frontal gyrus; L-MC/R-MC, the left/right motor cortex, which includes both the primary motor cortex (M1) and the pre-supplementary motor area (Pre-SMA); L-PSC/R-PSC, the left/right primary somatosensory cortex; L-OL/R-OL, the left/right occipital lobe cortex; t1, baseline time points; t2, post-therapy.

This study evaluated 40 Hz BBT music therapy versus conventional treatment using a parallel-group design. Participants (*n* = 42) were randomized 1:1 using an SPSS 26.0-generated sequence. Outcome assessors and statisticians remained blinded, whereas the participants/therapists were unblinded due to intervention specificity. Group allocation was performed using sequentially numbered opaque envelopes with unique identifiers. Both groups received standardized rehabilitation (matched intensity/frequency), and outcomes were assessed at baseline (t1) and post-intervention (t2). The flowchart of this study is shown in Figure 1.

### 2.3 Intervention

#### 2.3.1 Conventional arousal-promoting interventions

Both the 40 Hz BBT music therapy and control groups received identical daily conventional arousal-promoting interventions consisting of:

1. Median nerve stimulation (15 mA, right middle finger flexion, 4 h);
2. tDCS (1 mA, anode: affected hemisphere; 30 min);
3. Low-frequency rTMS (DLPFC-targeted, 8 Hz; 20 min);
4. Electroacupuncture (Renzhong/Neiguan/Yongquan acupoints; 30 min).

This standardized approach ensured the matching of both treatment intensity (identical stimulation parameters) and frequency (once-daily sessions) across all study participants.

#### 2.3.2 40 Hz BBT music therapy

To personalize music interventions and ensure therapeutic efficacy, certified music therapists conducted structured interviews with primary caregivers to select five emotionally and culturally significant compositions per patient. When preferences were unavailable, selections were made from our standardized music library (Yan et al., 2024), containing culturally representative pieces such as Spring Festival Overture and Summer (Joe Hisaishi).

To optimize the impact of the music intervention, binaural beats at 40 Hz were embedded. These were generated by presenting two pure sinusoidal tones of slightly different frequencies—250 Hz to the left ear and 290 Hz to the right ear—using Audacity software. This dichotic presentation exploits brainstem coincidence detection in the medial superior olive, where the interaural frequency disparity (Δf = 40 Hz) produces an endogenous frequency-following response, driving frequency-specific neural entrainment and engaging thalamocortical gamma oscillations. Unlike peripheral amplitude-modulated stimulation, this mechanism centralizes beat generation at the subcortical level and has been shown to modulate cognitive function. The music combined with binaural beats was played back using noise-canceling headphones, with the stimulation volume adjusted to 65 dB. The study termination criteria included: (1) disease progression posing a threat to the patient’s life; (2) mortality; and (3) occurrence of severe adverse reactions. These criteria were established to ensure patient safety and ethical compliance throughout the study.

The 40 Hz BBT music therapy protocol, initiated post-enrollment, comprised two daily 60-min sessions (7:30–8:30 a.m.; 4:30–5:30 p.m.) during patients’ alert states, avoiding interference with the implementation of concurrent therapies. The 16-day regimen consisting of 7 consecutive treatment days, followed by 2 rest days, and an additional 7 treatment days minimized auditory habituation while preserving therapeutic efficacy.

### 2.4 Outcome measures

All DOC patients underwent comprehensive baseline (t1) assessments, including CRS-R evaluation, EEG, and fNIRS. Identical multimodal evaluations were repeated post-therapy (t2) after a complete treatment cycle. EEG and fNIRS evaluations were conducted sequentially over 2 days within the same week under identical environmental and experimental conditions (including room setup, lighting, and acoustic stimulation protocol). In cases of transient physiological contraindications (e.g., low-grade fever >37.5 °C), neurophysiological assessments were postponed for 24 h to ensure patient safety and data reliability.

#### 2.4.1 EEG

##### 2.4.1.1 Data acquisition

EEG was recorded using a wireless 16-channel digital EEG system (ZN16E, Chengdu, China) configured according to the 10–20 international system. The 16 recording electrodes included FP1, FP2, F3, F4, C3, C4, P3, P4, O1, O2, F7, F8, T3, T4, T5, and T6, with linked earlobes serving as reference. Fz, Cz, and Pz were used as additional reference points during recording (Figure 1A). Signal digitization was performed at 500 Hz (bandwidth: 0.3–100 Hz). Throughout the recordings, subjects remained supine while a rehabilitation specialist monitored real-time EEG for signs of drowsiness (e.g., theta augmentation, K-complexes, spindle activity). To prevent sleep from confounding the resting-state data, brief deep pressure stimulation was applied only when vigilance decrements were detected—ensuring that all analyzed segments reflected a stable, wakeful baseline rather than a passive state contaminated by arousal fluctuations. To optimize signal integrity, the participants were instructed to minimize movement and maintain ocular closure throughout the sessions. All recordings underwent rigorous visual inspection by clinical neurophysiologists to exclude segments contaminated with electromyographic or oculomotor artifacts. Subsequent offline analysis was performed using MATLAB-based processing pipelines. Artifact-laden epochs were excluded using manual verification. Finally, 60 artifact-free resting-state segments (65.536 s/segment; 32,768 data points) were selected per subject. Spectral filtering included a 0.3 Hz high-pass filter to remove slow drift, a 70 Hz low-pass filter to attenuate myogenic noise, and a 50 Hz notch filter to eliminate line interference.

##### 2.4.1.2 Data analysis

###### 2.4.1.2.1 ApEn

Building upon Pincus’ seminal work (Pincus, 2000), we employed approximate entropy (ApEn) as a rigorously validated measure of signal complexity, providing a nonlinear dynamic assessment of cortical information processing. This metric quantifies the conditional probability of pattern recurrence within a physiological time series, with lower values indicating greater system regularity and higher values indicating increased complexity (Sarà et al., 2011). The ApEn calculation incorporated three critical parameters, including the length of the elapsed time ( N ), the predicted subsequent value ( m ), and the filtering level of ApEn ( r ). To improve the accuracy of the analysis, N was fixed at 4096. ApEn was calculated as follows:

$$\begin{array}{l} \text{ApEn}(m,r,L)=\frac{1}{L-m}\sum_{i=1}^{L-m}\log C_{i}^{m+1}(r)\\ -\frac{1}{L-m+1}\sum_{i=1}^{L-m+1}\log C_{i}^{m}(r) \end{array}$$ (1)

Where m is set to 2 and r =0.15% × SD (SD is the standard deviation of the original time series XN ), the pattern length ( m ) was fixed at 2 s. Regional ApEn values were calculated by averaging across six standard EEG-derived cortical regions: prefrontal lobe (FP1-FP2 mean), frontal (F3-F4 mean), central (C3-C4 mean), parietal (P3-P4 mean), occipital (O1-O2 mean), and temporal (F7-F8-T3-T4-T5-T6 mean) regions.

###### 2.4.1.2.2 C-ApEn

Cross-approximate entropy (C-ApEn) serves as a robust metric for assessing asynchrony between paired neurophysiological signals (Zhang et al., 2021). As a bivariate statistical measure, C-ApEn extends beyond single-signal analysis to quantify the interaction complexity between distinct brain regions. The computation requires two concurrent time series, each of length N , with the mathematical formulation as follows:

$$\phi^{m}(r)(v\|u)=\frac{1}{N-m+1}\sum_{i=1}^{N-m+1}\log C_{i}^{m}(r)(v\|u)$$ (2)

$$\begin{array}{l} \text{Cross}-\text{ApEn}\;\;(M,R,N)(v\|u)=-\phi^{m}(r)(v\|u)\\ -\phi^{m+1}(r)(v\|u), \end{array}$$ (3)

The electrode pairing protocol for C-ApEn analysis was implemented as follows: C3-P3, C3-F3, C3-T3, C3-FP1, C3-O1, C4-P4, C4-F4, C4-T4, C4-FP2, C4-O2, T5-F7, C3-F7, T6-F8, and C4-F8. Regional C-ApEn measures were subsequently derived through the following composite metrics: Central-parietal: [(C3-P3) + (C4-P4)]/2, Central-frontal: [(C3-F3) + (C4-F4)]/2, Central-middle temporal: [(C3-T3) + (C4-T4)]/2, Central-prefrontal lobe: [(C3-FP1) + (C4-FP2)]/2, Central-anterior temporal: [(C3-F7) + (C4-F8)]/2, Central-occipital: [(C3-O1) + (C4-O2)]/2.

#### 2.4.2 fNIRS

##### 2.4.2.1 Data acquisition

The study utilized a continuous-wave near-infrared spectroscopy system (NirSmart-6000A, Danyang Huichuang Medical Equipment Co., Ltd., China) with dual-wavelength (730 and 850 nm) illumination at a sampling rate of 11 Hz. A total of 24 light source probes and 16 detection probes were used in the experiment, totaling 48 effective channels, with an average distance of 3 cm (range 2.7–3.3 cm) between the emitters and detectors. The channel locations were calibrated to the Brodmann cerebral cortex partitions. Based on the coordinates, the 46 channels were divided into 12 regions of interest (ROIs) corresponding to the left and right hemispheres of the participants’ brains (Figure 1B).

Specifically, these ROIs include the left/right dorsolateral prefrontal cortex (L-DLPFC-CH 23, 25; R-DLPFC-CH 17, 18), left/right prefrontal cortex (L-PFC-CH: 8, 9, 10, 22, 24; R-PFC-CH 5, 6, 7, 19, 21), left/right inferior frontal gyrus (L-IFG-CH 11, 12; R-IFG-CH 3, 4), left/right motor cortex (L-MC-CH 13, 14, 26, 27, 31, 43, 44; R-MC-CH 1, 2, 16, 28, 29, 42, 45), which includes both the primary motor cortex (M1) and the pre-supplementary motor area (Pre-SMA), left/right primary somatosensory cortex (L-PSC-CH 30, 32, 33; RPSC-CH 34, 35, 15), and left/right occipital lobe cortex (L-OL-CH 40, 41, 39, 47; ROL-CH 37, 38, 46, 36). Due to the limited number of channels in this study, the motor cortex (MC) encompasses both the primary motor cortex (M1) and the pre-supplementary motor area (Pre-SMA). Channels (CH 48 and CH 20) that could not be clearly distinguished between the left and right hemispheres were excluded (Figure 1B). A detailed mapping of each of the 46 channels to these 12 ROIs, along with corresponding MNI coordinates and Brodmann areas, is provided in Supplementary Table 1.

**Figure 2:**
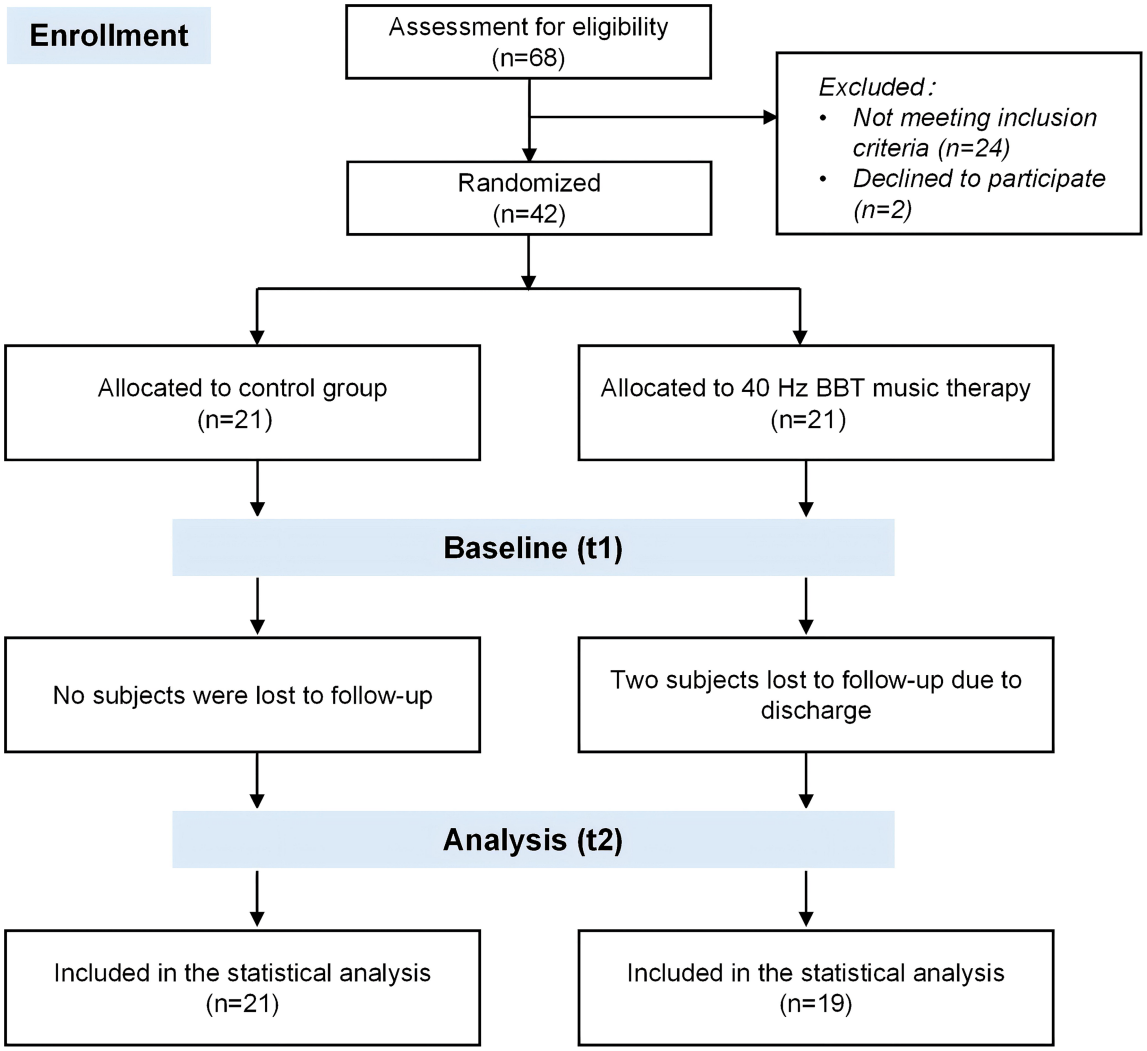
Study flow diagram of patient enrollment and allocation. BBT, binaural beat therapy; t1, baseline time points; t2, post-therapy.

##### 2.4.2.2 Data processing

The original data were processed using Matlab-based NirSpark software (version 1.7.5) developed in Danyang, China. The original data consisted solely of light-intensity signals and contained significant noise. Before the signals passed through the filter, we further inspected and removed noisy channels with obvious abnormal fluctuations based on our experience. The data processing steps were as follows: (1) automated removal of motion artifacts caused by head movements; (2) conversion of light intensity signals to optical density signals; (3) application of a bandpass filter (0.01 Hz < *f* < 0.2 Hz) to eliminate physiological noise (heartbeat within 1–1.5 Hz, respiration within 0.2–0.5 Hz) and low-frequency drift (<0.09 Hz); (4) subsequently, the modified Beer–Lambert law with a differential path length factor of 6 for each wavelength was employed to convert optical density data into concentrations of oxygenated hemoglobin (HbO).

##### 2.4.2.3 Functional connectivity analysis

We adopted a functional connectivity (FC) analysis method to observe the impact of music therapy on cortical network functional connectivity. Pearson’s correlation coefficient was used to examine the functional connections between each pair of measurement channels. Thus, a 12 × 12 correlation matrix was generated for each participant. However, the correlation values (*R*-values) cannot be directly added; therefore, Fisher’s *r*-to-*z* transformation was used to convert the *R*-values to *z*-values. The *z*-value was defined as the strength of the FC between channels to calculate the average correlation value between ROIs. A significance level of *p* < 0.05 was considered significant.

#### 2.4.3 Primary endpoints

1. EEG-derived complexity metrics (ApEn, C-ApEn);
2. fNIRS-derived average FC of HbO;
3. CRS-R total and subscale scores.

### 2.5 Statistical analysis

Baseline demographic characteristics were compared using one-way ANOVA for continuous variables (normality confirmed by Shapiro–Wilk tests, *p* > 0.05) and chi-square tests for categorical variables, with all analyses performed on admission data to ensure group comparability. Within-group pre-post changes in neurobehavioral (CRS-R scores), entropy (ApEn/C-ApEn), and hemodynamic (HbO connectivity) outcomes were analyzed using paired *t*-tests, and normality was confirmed using Shapiro–Wilk tests (all *p* > 0.05). Cohen’s *d* was calculated to quantify the effect size of within-group changes.

Independent-samples Student’s *t*-tests were used to compare the pre-to-post treatment change scores (*Δ* = t2 − t1) between the control group (*n* = 19) and the 40 Hz BBT music therapy group (*n* = 21). Specifically, CRS-R total and subscale scores were analyzed for the overall sample, while EEG metrics (ΔApEn, ΔC-ApEn) and ΔFC of HbO were evaluated both for the overall sample and stratified by consciousness status (VS/UWS and MCS subgroups)—this stratified analysis aimed to account for disease severity differences and clarify subgroup-specific treatment-related changes in neurophysiological outcomes. Cohen’s *d* was calculated for all comparisons to quantify the effect size of between-group differences in treatment response.

To assess the association between clinical improvement and neurophysiological changes, a within-subject change-score correlation analysis was conducted. This analysis examined the relationships between changes in clinical status (ΔCRS-R) and changes in each of the aforementioned neurophysiological markers (ΔApEn, ΔC-ApEn, ΔHbO-FC). Pearson correlation coefficients were further calculated to evaluate the linear relationships between ΔCRS-R and these three neurophysiological change scores. All tests were two-tailed, with statistical significance set at *p* < 0.05.

## 3 Results

### 3.1 Demographic and clinical characteristics of participants

As shown in Figure 2, 42 patients were initially screened at t1. During treatment, two patients were excluded (one due to ICU transfer for clinical deterioration, and one due to muscle rigidity affecting assessment validity). The final analysis (t2) included 21 patients who received 40 Hz BBT music therapy and 19 in the control group.

Table 1 summarizes the baseline characteristics of the 40 patients with DOC who completed the trial. In the control group, the etiologies of DOC included traumatic brain injury (TBI) in two cases and non-TBI in 17 cases. The 40 Hz BBT music therapy group consisted of two TBI cases and 19 nTBI cases. The cohort comprised 11 males and 29 females (control group: 4 males, 15 females; 40 Hz BBT group: 7 males, 14 females). The mean age of the entire cohort was 60.8 ± 13.5 years (control: 60.21 ± 14.29; 40 Hz BBT: 61.24 ± 12.83), with medians of 60 and 64 years, respectively. Demographic and clinical characteristics, including age, time since injury, baseline CRS-R scores, sex distribution, and the proportion of MCS patients, did not differ significantly between the two groups (*p* > 0.05). Importantly, no adverse events attributable to the study interventions were reported during the trial period.

**Table 1:** Baseline demographic characteristics.

Characteristic	Control group	40 Hz BBT music therapy
	*n* = 19	*n* = 21
Male (*n*, %)	4, 21%	7, 33%
Age (years)	60.21 ± 14.29	61.24 ± 12.83
Cause of injury (TBI/stroke/HIE)	2/16/1	2/17/2
Time since injury (days)	144.84 ± 124.92	89.67 ± 56.57
Lesion lateral (left/right/both)	2/3/14	4/4/13
CRS-R scores	5.00 ± 2.71	5.29 ± 2.81

BBT, binaural beat therapy; CRS-R: Coma Recovery Scale-Revised; TBI, traumatic brain injury; HIE, hypoxic–ischemic encephalopathy.

### 3.2 Post-treatment CRS-R scores and changes in consciousness states

**Figure 3:**
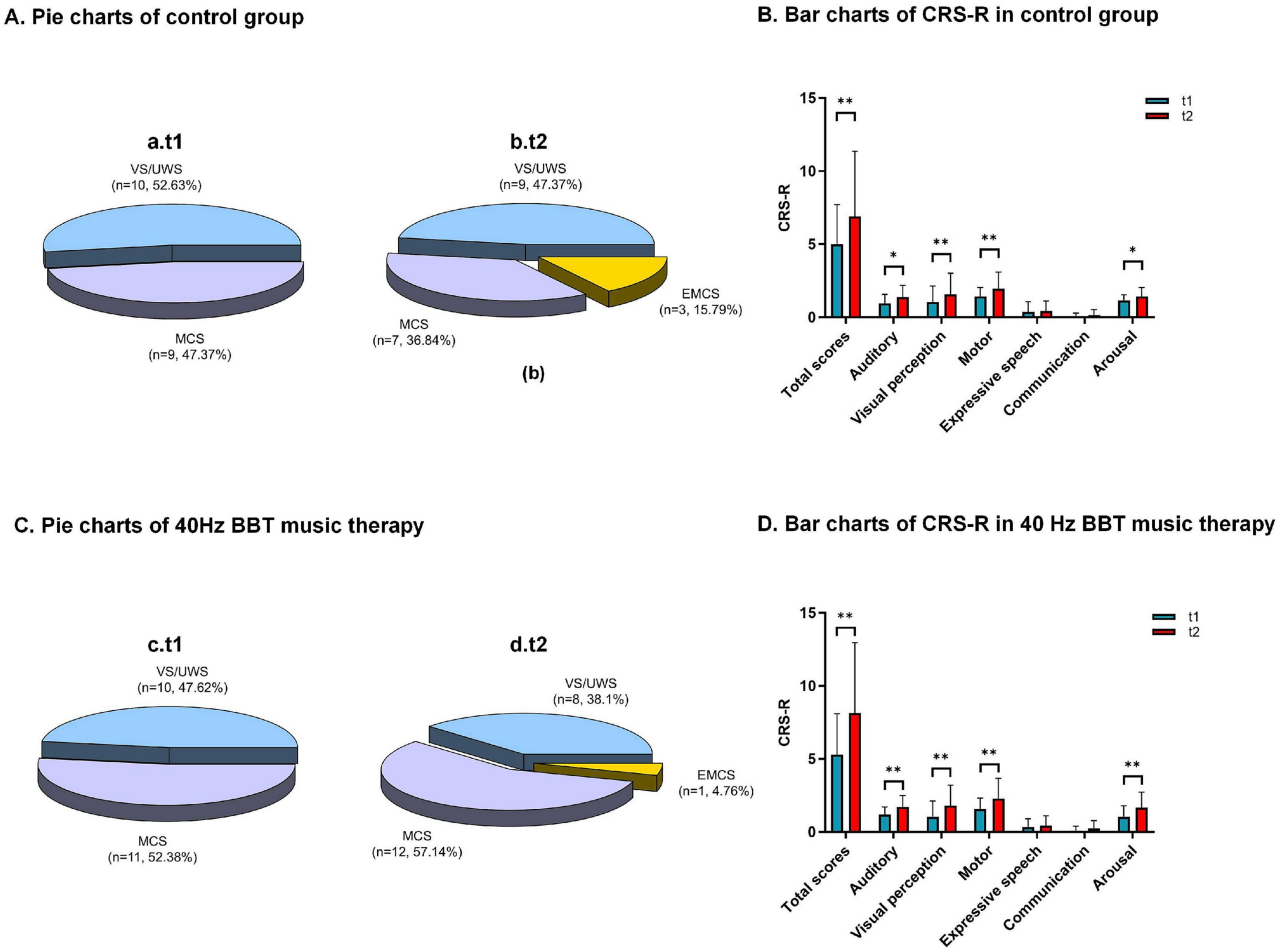
Changes in consciousness state in patients with DOC before and after treatment **(A)** Pie charts of the control group at t1 **(a)** and t2 **(b)**. **(B)** Bar charts of CRS‑R scores in the control group. **(C)** Pie charts of the 40‑Hz BBT group at t1 **(c)** and t2 **(d)**. **(D)** Bar charts of CRS‑R scores in the 40‑Hz BBT group.

Serial CRS-R assessments revealed significant treatment-related improvements in consciousness metrics (Supplementary Table 2). In the 40 Hz BBT music therapy group, total CRS-R scores increased from 5.29 ± 2.81 to 8.14 ± 4.82 (*Δ* = 2.86 ± 3.72, Cohen’s *d* = 0.77, *p* < 0.01), while the control group showed a smaller increase from 5.00 ± 2.71 to 6.89 ± 4.46 (*Δ* = 1.89 ± 2.28, p < 0.01). Significant improvements were observed across auditory, visual, motor, and arousal subscales in both groups (*p* < 0.05). Notably, the arousal subscale exhibited the largest effect size in the 40 Hz BBT music therapy group (*Δ* = 0.62 ± 0.67, Cohen’s *d* = 0.93, *p* < 0.01), compared with a moderate gain in controls (*Δ* = 0.26 ± 0.45, *p* < 0.01). Consciousness state transitions manifest through two distinct pathways: progression from VS/UWS to the MCS and subsequent advancement from MCS to emergence from MCS (EMCS), details are presented in the pie chart of Figure 3.

In addition, ΔCRS-R in most subscales showed no significant differences between groups, whereas ΔCRS-R in the arousal subscale approached marginal significance (*p* = 0.06, Cohen’s *d* = 0.62), favoring the 40 Hz BBT music therapy (Supplementary Table 2). This suggests a potential specific benefit on arousal regulation with the 40 Hz intervention, despite no significant between-group difference in the overall ΔCRS-R total score (*p* = 0.33).

### 3.3 Effects on brain function after treatment

**Figure 4:**
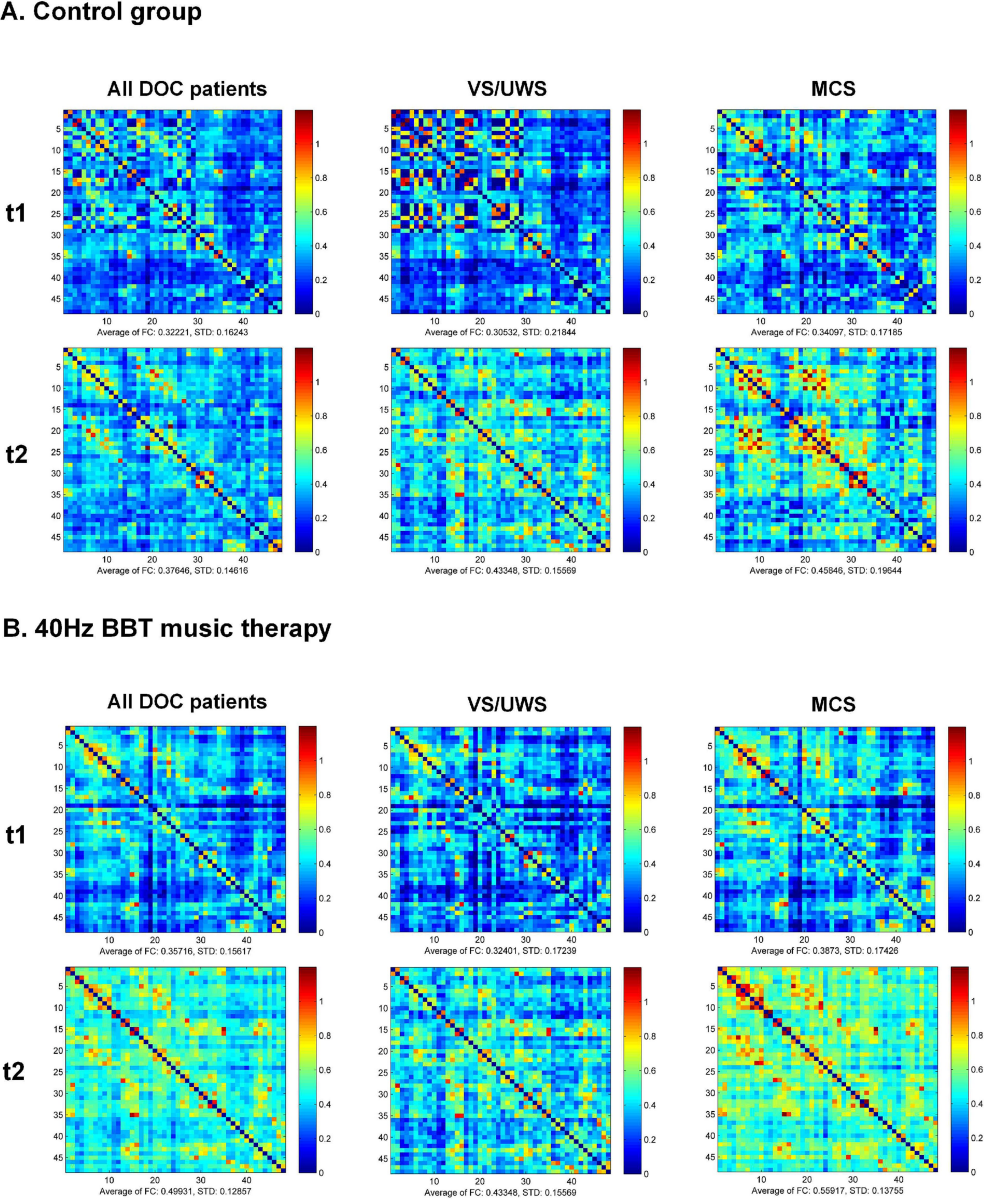
Changes in FC matrices in patients with DOC before and after treatment **(A)** Control group FC matrices at t1 and t2. **(B)** 40 Hz BBT group FC matrices at t1 and t2. Average FC and STD are shown for all DOC patients, VS/UWS, and MCS subgroups.

**Figure 5:**
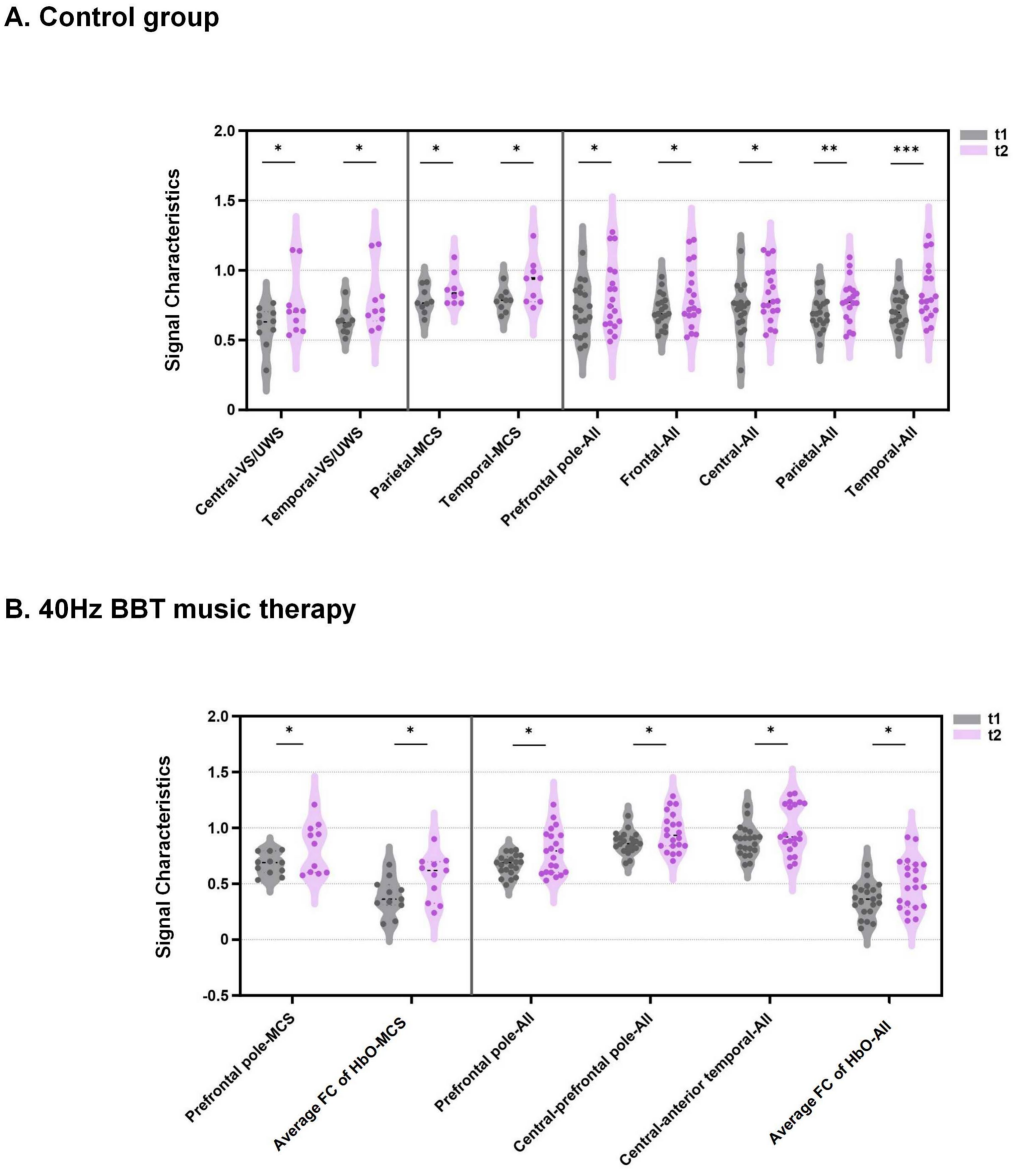
Violin plots of significant differences in FC, ApEn, and C-ApEn. Pre- (t1, gray) and post-treatment (t2, purple) comparisons of EEG entropy and fNIRS HbO functional connectivity in the control **(A)** and 40 Hz-BBT music therapy **(B)** groups. Violin plots show the distribution of values for each brain region/connectivity metric, with individual data points overlaid. Significance levels are denoted as: ^*^p < 0.05, ^**^p < 0.01, ^***^p < 0.001. BBT, binaural beat therapy; ApEn, approximate entropy; C-ApEn, cross-approximate entropy; FC, functional connectivity; MCS, minimally conscious state; VS/UWS, vegetative state/unresponsive wakefulness syndrome; t1, baseline time points; t2, post-therapy.

Longitudinal changes in EEG and fNIRS biomarkers are presented in Supplementary Table 3; Supplementary Table 4; Figures 4, 5. In the control group, significant increases in ApEn were observed in prefrontal pole (*p* = 0.043, Cohen’s *d* = 0.50), frontal (*p* = 0.046, Cohen’s *d* = 0.49), central (*p* = 0.016, Cohen’s *d* = 0.61), parietal (*p* = 0.006, Cohen’s *d* = 0.72), and temporal regions (*p* < 0.001, Cohen’s *d* = 0.94). Subgroup analysis revealed increased ApEn in VS/UWS patients (central: Cohen’s *d* = 0.81; temporal: Cohen’s *d* = 0.82) and MCS patients (parietal: Cohen’s *d* = 0.88; temporal: Cohen’s *d* = 1.21). No significant changes were observed in C-ApEn or average FC of HbO (all *p* > 0.05).

In the 40 Hz BBT music therapy group, significant increases were found in prefrontal pole ApEn (*p* = 0.007, Cohen’s *d* = 0.65), central-prefrontal pole C-ApEn (*p* = 0.019, Cohen’s *d* = 0.56), central-anterior temporal C-ApEn (*p* = 0.036, Cohen’s *d* = 0.49), and average FC of HbO (p < 0.001, Cohen’s *d* = 0.86). Subgroup analysis showed effects were driven by MCS patients (prefrontal ApEn: Cohen’s *d* = 0.70; average FC of HbO: Cohen’s *d* = 1.11), with no significant changes in VS/UWS patients of 40 Hz BBT music therapy (all *p* > 0.05).

Between-group comparisons (Supplementary Table 4) revealed that the control group showed greater improvements in parietal (*p* = 0.012) and occipital (*p* = 0.016) ApEn in VS/UWS patients, occipital ApEn in MCS patients (*p* = 0.015), and central-occipital C-ApEn in MCS patients (p = 0.007), with a trend toward greater average FC of HbO improvement overall (*p* = 0.047).

**Figure 6:**
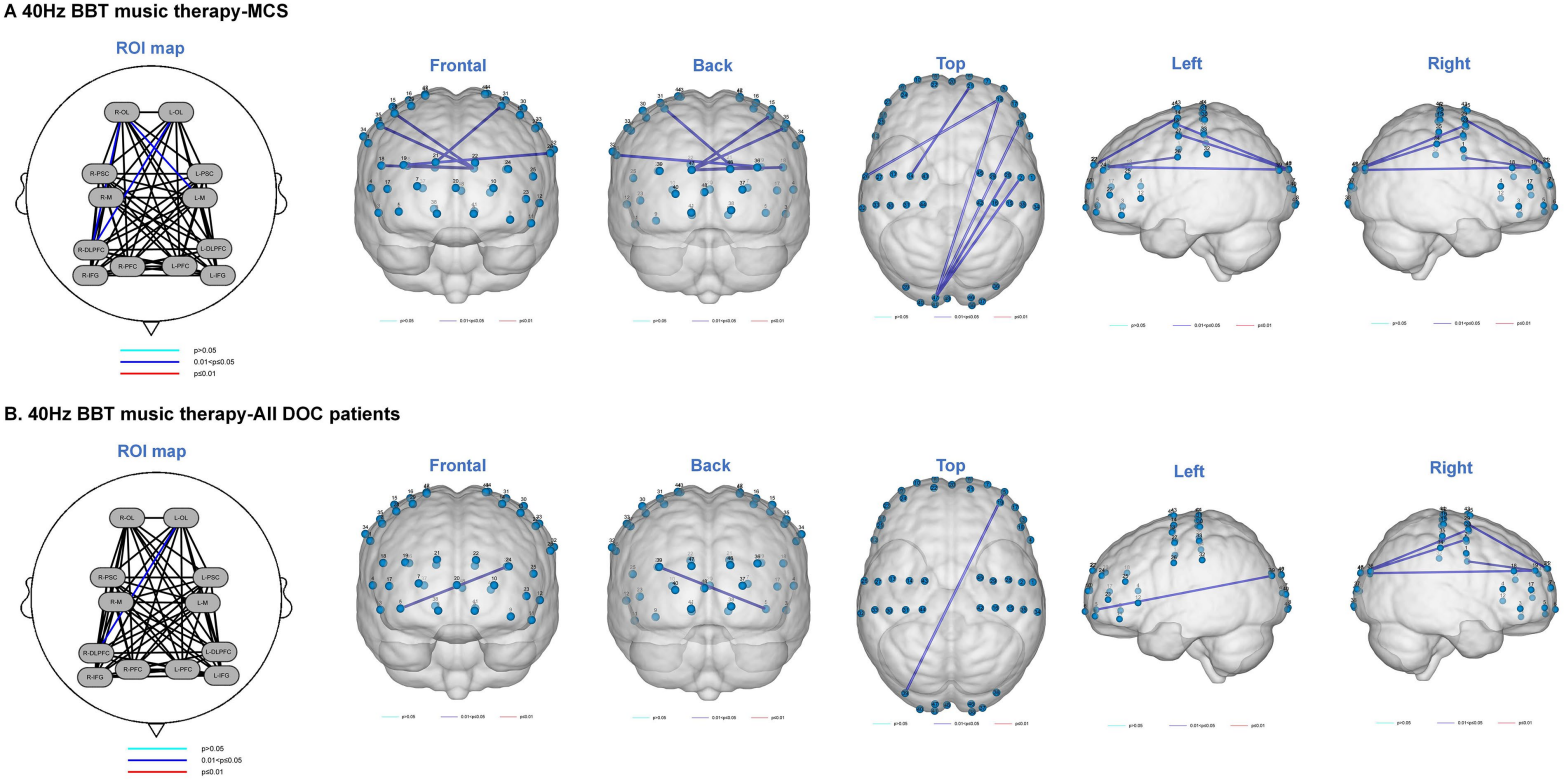
Brain network connectivity with significant differences (FDR‑corrected) **(A)** 40 Hz BBT – MCS subgroup. **(B)** 40 Hz BBT – all DOC patients. Significant increases (t2 vs. t1) were observed in both; no significant differences in VS/UWS or control group.

FC mapping (false discovery rate, FDR-corrected) further delineated subgroup-specific reorganization (Figure 6): MCS patients showed enhanced connectivity in three key pathways (R-DLPFC ↔ R/L-OL, R-OL ↔ L-MC); the overall DOC group exhibited selective R-DLPFC ↔ R-OL strengthening; VS/UWS patients in the 40 Hz BBT music therapy group showed no significant electrophysiological or connectivity changes.

### 3.4 Correlation between CRS-R scores and EEG/fNIRS metrics

**Figure 7:**
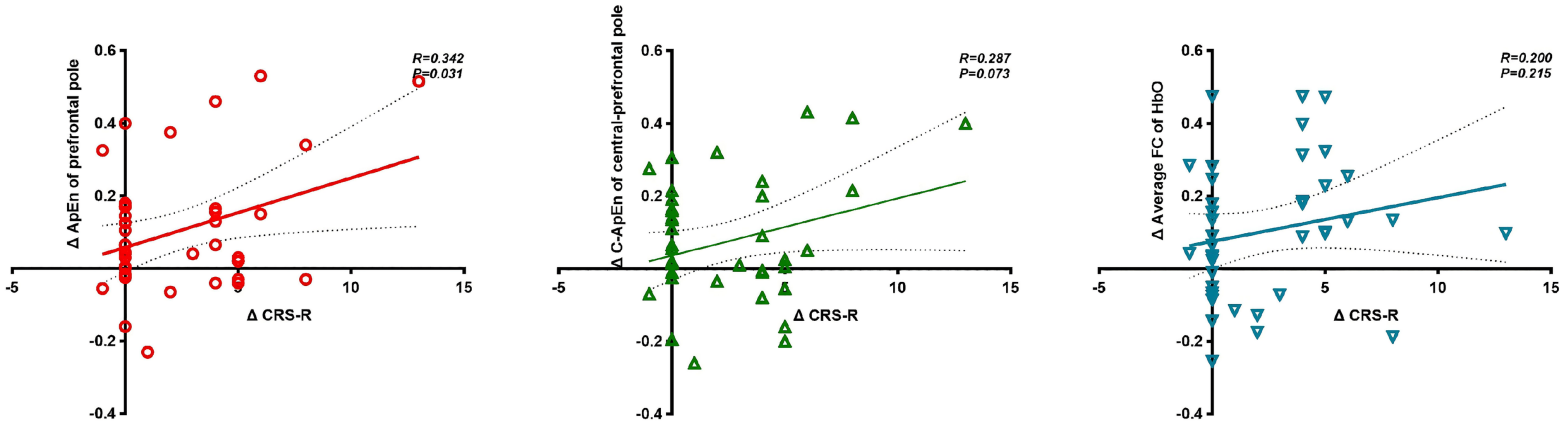
Correlations between *Δ*CRS-R and ΔApEn, ΔC-ApEn, and ΔFC. *Δ*, change from baseline (t1) to post-intervention (t2). BBT, binaural beat therapy; C-ApEn, causal approximate entropy; CRS-R, Coma Recovery Scale–Revised; ApEn, approximate entropy; FC, functional connectivity; t1, baseline time points; t2, post-therapy.

Correlation analyses revealed that improvements in CRS-R scores (ΔCRS-R) were positively correlated with ΔApEn of prefrontal pole (*R* = 0.324, *p* = 0.031, two-tailed), indicating that increased prefrontal signal complexity was associated with greater clinical recovery (Figure 7). A non-significant trend was observed for ΔC-ApEn of central-prefrontal pole region (*R* = 0.287, *p* = 0.073), while Δaverage FC of HbO showed a weak, non-significant correlation (*R* = 0.200, *p* = 0.215). These findings suggest that electrophysiological changes in prefrontal cortical dynamics are associated with consciousness improvement following 40 Hz BBT music therapy, whereas hemodynamic connectivity changes show a weaker correlation with clinical recovery.

## 4 Discussion

Unlike our prior diagnostic study based on single-session EEG (Qu et al., 2024), the present study introduces a novel, repeated-measures multimodal paradigm. This approach integrates complementary EEG and fNIRS across pre- and post-intervention assessments to systematically uncover longitudinal neurophysiological dynamics. This study revealed that 40 Hz BBT combined with preferred music differentially modulates neural dynamics in patients with DOC. Key findings include: (1) Patients in an MCS, but not a VS/UWS, exhibited increased ApEn of the prefrontal pole and average FC of HbO following intervention. (2) Significant correlations emerged between these neural biomarkers and CRS-R scores, particularly for ΔApEn of the prefrontal pole (*R* = 0.324, *p* = 0.031), indicating their biomarker potential for consciousness recovery. These findings advance the development of personalized frequency-specific neuromodulation for DOC.

### 4.1 Non-invasive 40 Hz neuromodulation: combining preferred music and BBT

Extensive research has established that music engages self-awareness substrates even in severe cognitive impairment, where melody modulates mesolimbic dopamine, while rhythm recruits corticomotor pathways (Kotchoubey et al., 2015). Although α-band BBT-enhanced music improves DOC responsiveness (Liu C. et al., 2022; Liu Z. B. et al., 2022) and gamma binaural beats enhance Alzheimer’s cognition (Liu C. et al., 2022; Liu Z. B. et al., 2022), our study makes a unique contribution by demonstrating the neural benefits of 40 Hz-BBT music therapy in patients with MCS via multimodal assessment. The 40 Hz stimulation enhances consciousness by entraining gamma oscillations, synchronizing thalamocortical networks, and optimizing interneural communication (Binder et al., 2017). Relevant human literature further supports the pro-cognitive effects of 40 Hz binaural beats. For instance, Fujikawa et al. (2025) showed that inaudible 40 Hz binaural beats significantly reduced reaction times in an attention network test, suggesting improved attentional efficiency. Ross and Lopez (2020) demonstrated that 40 Hz binaural beat stimulation accelerated training outcomes in an attentional blink task, with performance gains emerging after sleep consolidation. Additionally, Sharpe et al. (2020) reported that 40 Hz entrainment improved memory scores (from 87 to 95%, *p* = 0.0027) and showed a trend toward cognitive enhancement in healthy participants. From a mechanistic perspective, 40 Hz BBT music therapy combines: (1) music-driven activation of emotion/memory networks (enhancing plasticity) (Qu et al., 2024; Castro et al., 2015; Qu et al., 2024; Castro et al., 2015); and (2) brainstem-mediated BBT propagation (superior olivary complex → reticular formation → thalamocortical circuits; Engelbregt et al., 2021; Kim et al., 2023; Beauchene et al., 2016; Garcia-Argibay et al., 2019; Ingendoh et al., 2023), synchronizing interhemispheric activity and optimizing functional connectivity. Notably, preclinical work by Wang et al. (2023) demonstrated that 40 Hz gamma entrainment rescued cognitive impairment in a mouse model of traumatic brain injury, supporting the neuroprotective and neuromodulatory potential of gamma-frequency stimulation. These findings provide further clinical evidence that external noninvasive auditory stimulation can facilitate neural modulation and may support cognitive and neural recovery.

### 4.2 Post-treatment changes in cortical complexity

DOC arise from disrupted neural networks, primarily marked by reduced complexity and functional connectivity (Sarà et al., 2011). These impairments, in turn, lead to cortical isolation and disorganized behavioral responses (Ferenets et al., 2006). Consistent with previous research, our findings confirm that prefrontal lobe complexity (ApEn) strongly correlates with CRS-R scores (*p* < 0.05), thereby supporting the established evidence linking nonlinear EEG metrics to DOC severity (Sarà et al., 2011). Notably, key observations revealed treatment-induced ApEn increases across multiple regions (prefrontal lobe, frontal, central, parietal, and temporal) in all DOC patients. In comparison, the control group exhibited spontaneous complexity changes, potentially reflecting preserved neuroplasticity, particularly in patients with a shorter disease duration (<1.5 years) (Liu C. et al., 2022; Liu Z. B. et al., 2022). These elevated ApEn values suggest a transition from synchronized, low-activity states to desynchronized, high-complexity patterns (Liu et al., 2021), indicating compensatory reorganization of the brain.

In contrast, the 40 Hz-BBT music therapy demonstrated a distinct and significant increase in ApEn values, specifically in the prefrontal pole, highlighting the positive influence of music therapy on brain function in patients with DOC. Importantly, this effect was more pronounced in patients with MCS, with no significant changes observed in patients with VS/UWS. Given that the prefrontal pole region is crucial for higher-order cognitive functions and consciousness integration (Mizrahi and Axelrod, 2023), increased ApEn values may reflect enhanced neural activity complexity driven by gamma-frequency (40 Hz) entrainment. In patients with MCS, 40 Hz-BBT music therapy may activate residual consciousness-related networks and facilitate partial restoration of brain function. Conversely, patients with VS/UWS, who typically exhibit extensive structural damage and severely disrupted network connectivity (Porcaro et al., 2021; Chen et al., 2018), show limited enhancement in neural activity complexity in the prefrontal pole region, suggesting limited responsiveness to auditory-based neuromodulation.

### 4.3 Post-treatment changes in cross-regional electrophysiological coordination

Combining C-ApEn (EEG-based cross-regional complexity) revealed network impairments in DOC patients. Importantly, the control group showed no improvement in this measure, indicating that conventional treatments may fail to restore cross-regional electrophysiological coordination in chronic, low-activity states (Thibaut et al., 2021).

In the intervention group, 40 Hz BBT music therapy induced several distinct network-level improvements. First, we observed significantly increased C-ApEn values in the prefrontal-central-temporal regions (all *p* < 0.05), indicating enhanced local microstate diversity and improved interregional communication. This effect likely reflects the intervention’s ability to boost gamma-band synchronization between key cognitive regions, specifically the prefrontal cortex (responsible for higher-order functions) and anterior temporal lobe (involved in memory/emotion processing) (Helfrich and Knight, 2019). Such enhanced synchronization may consequently optimize the overall network efficiency (Liu et al., 2013). Of particular note, a non-significant trend was observed for the correlation between ΔC-ApEn of the central-prefrontal region and CRS-R improvements (*R* = 0.287, *p* = 0.073), whereas ΔApEn of the prefrontal pole showed a significant correlation with clinical recovery (*R* = 0.324, *p* = 0.031). This distinction indicates that, in the present study, regional prefrontal signal complexity was more closely associated with consciousness improvement following 40 Hz BBT music therapy than was cross-regional electrophysiological coordination.

### 4.4 Post-treatment changes in functional connectivity

At the hemodynamic level, fNIRS data revealed differential responses between the diagnostic subgroups. In patients with MCS, 40 Hz BBT music therapy induced maximal network reorganization, characterized by significantly enhanced connectivity between the right DLPFC and bilateral OL (*p* < 0.01), as well as between the ROL and LMC (*p* < 0.05), findings that align well with established mechanisms of consciousness recovery (Chen et al., 2018; Thibaut et al., 2021). In stark contrast, VS/UWS patients showed no detectable connectivity changes (all *p* > 0.1), thus revealing a clear neurophysiological severity gradient across DOC subtypes.

In the context of 40 Hz BBT music therapy, comparison of neuroimaging modalities revealed that ΔApEn of the prefrontal pole showed a significant correlation with CRS-R improvements (*R* = 0.324, *p* = 0.031), whereas Δaverage FC of HbO exhibited a weak, non-significant correlation (R = 0.200, *p* = 0.215). This pattern suggests that the therapeutic effects of 40 Hz BBT music therapy on consciousness recovery may be more closely reflected by electrophysiological changes in prefrontal cortical dynamics than by hemodynamic connectivity changes. The weak correlation between Δaverage FC of HbO and CRS-R improvements may be attributed to several factors: the different temporal scales of hemodynamic versus electrophysiological signals, limited statistical power due to sample size, the use of global averaging that may obscure regionally specific patterns (e.g., the subgroup-specific connectivity reorganization observed in MCS patients), and patient heterogeneity across VS/UWS and MCS subgroups. These findings provide empirical support for the role of prefrontal cortical activity in mediating the therapeutic effects of 40 Hz BBT music therapy.

This study had five key limitations. First, the restricted sample size permitted only 40 Hz-BBT versus control comparisons; future larger trials should integrate four arms (preferred music-only, BBT-only, combined therapy, control) to dissect modality-specific and synergistic effects. Second, shorter intervention cycles may mask dose–response relationships, necessitating extended protocols with subgroup stratification (e.g., etiology-specific and EEG-profile-based cohorts) to refine neuromodulation precision. Third, the lack of longitudinal follow-up limits insight into sustained recovery; multi-timepoint assessments (e.g., 3/6/12-month) are critical to map consciousness trajectories and identify therapeutic windows. Fourth, while this study demonstrates that 40 Hz BBT combined with preferred music modulates neural activity in MCS patients, the absence of an alternative-frequency BBT control (e.g., 15 Hz) limits conclusions about frequency specificity. Although both groups received 20 Hz rTMS as part of conventional treatment, future studies should include active BBT controls at different frequencies to dissociate gamma-band effects from non-specific auditory stimulation. Finally, this study did not specifically assess DOC subgroups such as cognitive motor dissociation (Schiff, 2015), preserved auditory localization (Carrière et al., 2020), or later recovery of language function (Aubinet et al., 2019). Thus, the applicability of our findings to these populations remains uncertain. Future studies may also integrate EEG-fNIRS with whole-brain computational approaches, such as intrinsic ignition, to better understand recovery mechanisms in DOC (Panda et al., 2023).

## 5 Conclusion

The 40 Hz BBT combined with preferred music was associated with improvements in consciousness in patients with DOC, with relatively greater effects observed in those in a MCS. The findings suggest that changes in prefrontal electrophysiological dynamics, particularly increased signal complexity, may be more closely related to clinical improvement than hemodynamic connectivity measures. Overall, these results indicate that 40 Hz BBT combined with preferred music may have potential as a noninvasive supportive intervention, and that neurophysiological biomarkers may help to objectively assess treatment-related changes in DOC. Nevertheless, given the absence of a control group, these findings should be regarded as preliminary, and further validation in larger, well-designed studies is needed before definitive conclusions can be drawn.

## Data Availability

The raw data supporting the conclusions of this article will be made available by the authors, without undue reservation.
