# Supplementary material for: Cortical processing modulation in DOC by preferred music-coupled 40 Hz binaural stimulation: an exploratory EEG-fNIRS study
Source: Front Psychol. 2026 Jul 13;17:1783416. doi: 10.3389/fpsyg.2026.1783416 (PMC13402131; doi:10.3389/fpsyg.2026.1783416)
Supplement: Supplementary file 1 [file Data_Sheet_1.PDF]

Supplementary Table 1.fNIRS Channel Assignment to Anatomical ROIs via MNI Coordinates

Channel ID	MNI Coordinates (mm)			Brodmann Area	ROI
	X	Y	Z		
CH1 (S1-D1)	62	-7	42	BA 6	RMC
CH2 (S1-D12)	54	-7	54	BA 6	RMC
CH3 (S2-D2)	55	41	-8	BA 47	RIFG
CH4 (S2-D7)	61	26	10	BA 47	RIFG
CH5 (S3-D2)	41	63	-7	BA 10	RPFC
CH6 (S3-D3)	14	72	-8	BA 10	RPFC
CH7 (S3-D8)	28	68	11	BA 10	RPFC
CH8 (S4-D3)	-12	72	-8	BA 10	LPFC
CH9 (S4-D4)	-38	63	-13	BA 10	LPFC
CH10 (S4-D9)	-26	70	10	BA 10	LPFC
CH11 (S5-D4)	-53	42	-15	BA 47	LIFG
CH12 (S5-D10)	-59	26	-1	BA 47	LIFG
CH13 (S6-D11)	-45	-5	62	BA 6	LMC
CH14 (S6-D15)	-32	-6	69	BA 6	LMC
CH15 (S7-D12)	45	-27	68	BA 3	RPSC
CH16 (S7-D13)	34	-27	73	BA 4	RMC
CH17 (S8-D2)	49	51	9	BA 46	RDLPFC
CH18 (S8-D7)	53	35	26	BA 46	RDLPFC
CH19 (S8-D8)	37	54	26	BA 10	RPFC
CH21 (S9-D8)	15	65	28	BA 10	RPFC
CH22 (S9-D9)	-13	66	28	BA 10	LPFC
CH23 (S10-D4)	-49	52	2	BA 46	LDLPFC
CH24 (S10-D9)	-37	58	24	BA 10	LPFC
CH25 (S10-D10)	-54	37	18	BA 46	LDLPFC
CH26 (S11-D5)	-66	-6	35	BA 6	LMC
CH27 (S11-D11)	-56	-7	53	BA 6	LMC
CH28 (S12-D12)	44	-5	63	BA 6	RMC
CH29 (S12-D13)	32	-6	69	BA 6	RMC
CH30 (S13-D11)	-47	-29	67	BA 3	LPSC
CH31 (S13-D15)	-35	-28	73	BA 4	LMC
CH32 (S14-D5)	-69	-31	38	BA 2	LPSC
CH33 (S14-D11)	-58	-28	54	BA 2	LPSC
CH34 (S15-D1)	66	-30	47	BA 2	RPSC
CH35 (S15-D12)	55	-28	58	BA 2	RPSC
CH36 (S16-D6)	35	-92	26	BA 19	ROL
CH37 (S17-D6)	24	-104	10	BA 18	ROL
CH38 (S17-D16)	16	-107	-5	BA 18	ROL
CH39 (S18-D14)	-35	-93	23	BA 19	LOL
CH40 (S19-D14)	-24	-105	7	BA 18	LOL
CH41 (S19-D16)	-13	-106	-7	BA 18	LOL
CH42 (S20-D13)	22	-27	77	BA 4	RMC

CH43 (S21-D15)	-20	-6	77	BA 6	LMC
CH44 (S22-D15)	-22	-28	77	BA 4	LMC
CH45 (S23-D13)	22	-4	76	BA 6	RMC
CH46 (S24-D6)	15	-101	25	BA 19	ROL
CH47 (S24-D14)	-12	-101	24	BA 19	LOL

Abbreviations: BA, Brodmann Area; ROI, region of interest; LDLPFC, left dorsolateral prefrontal cortex; RDLPFC, right dorsolateral prefrontal cortex; LPFC, left prefrontal cortex; RPFC, right prefrontal cortex; LIFG, left inferior frontal gyrus; RIFG, right inferior frontal gyrus; LMC, left motor cortex; RMC, right motor cortex; LPSC, primary somatosensory cortex; RPSC, primary somatosensory cortex; LOL, left occipital lobe; ROL, right occipital lobe.
