# Supplementary material for: Cortical processing modulation in DOC by preferred music-coupled 40 Hz binaural stimulation: an exploratory EEG-fNIRS study
Source: Front Psychol. 2026 Jul 13;17:1783416. doi: 10.3389/fpsyg.2026.1783416 (PMC13402131; doi:10.3389/fpsyg.2026.1783416)
Supplement: Supplementary file 2 [file Data_Sheet_2.PDF]

Supplementary Table 2. Pre- and Post-treatment Comparison of CRS-R Scores

	Control group			40 Hz BBT music therapy			
	Time points	Mean±SD	Δ (t2-t1)	Time points	Mean±SD	Δ (t2-t1)	P_2 value (cohens'*d*)
CRS-R scores	t1	5.00±2.71	1.89±2.28	t1	5.29±2.81	2.86±3.72	
	t2	6.89±4.46		t2	8.14±4.82		
	P_1 value (cohens'*d*)	**<0.01 (0.83)**		P value (cohens'*d*)	**<0.01 (0.77)**		0.33 (0.31)
Auditory	t1	0.95±0.62	0.42±0.77	t1	1.19±0.51	0.52±0.87	
	t2	1.37±0.83		t2	1.71±0.78		
	P_1 value (cohens'*d*)	**0.03 (0.55)**		P value (cohens'*d*)	**0.01 (0.60)**		0.70 (0.12)
Visual perception	t1	1.05±1.08	0.53±0.77	t1	1.05±1.07	0.76±1.22	
	t2	1.58±1.43		t2	1.81±1.40		
	P_1 value (cohens'*d*)	**0.01 (0.68)**		P value (cohens'*d*)	**0.01 (0.62)**		0.48 (0.22)
Motor	t1	1.42±0.61	0.53±0.77	t1	1.57±0.75	0.71±1.06	
	t2	1.95±1.13		t2	2.29±1.38		
	P_1 value (cohens'*d*)	**0.01 (0.68)**		P value (cohens'*d*)	**0.01 (0.68)**		0.53 (0.19)
Expressive speech	t1	0.37±0.68	0.05±0.23	t1	0.33±0.58	0.10±0.30	
	t2	0.42±0.69		t2	0.43±0.68		
	P_1 value (cohens'*d*)	0.33 (0.23)		P value (cohens'*d*)	0.16 (0.32)		0.62 (0.18)
Communication	t1	0.05±0.23	0.11±0.32	t1	0.10±0.30	0.14±0.48	
	t2	0.16±0.37		t2	0.24±0.54		
	P_1 value (cohens'*d*)	0.16 (0.33)		P value (cohens'*d*)	0.19 (0.30)		0.77 (0.07)
Arousal	t1	1.16±0.37	0.26±0.45	t1	1.05±0.74	0.62±0.67	
	t2	1.42±0.61		t2	1.67±1.06		
	P_1 value (cohens'*d*)	**0.02 (0.58)**		P value (cohens'*d*)	**<0.01 (0.93)**		0.06 (0.62)

P_1 values were derived from paired t-tests, comparing pre-treatment (t1) and post-treatment (t2) scores within each group.

P_2 values were derived from two-tailed independent-samples Student's t-tests, comparing the change scores (Δ= t2-t1) between the two groups.

Cohen's *d* values represent the effect size for the corresponding comparisons.

Abbreviations: CRS-R: Coma Recovery Scale-Revised; Bold text indicates statistically significant differences between post-therapy (t2) and baseline (t1) measurements: **P<0.05** (two-tailed paired Student's t-tests for the six CRS-R subscales).

Supplementary Table 3. Pre- and Post-treatment Comparison of EEG and fNIRS Biomarkers

		Control group			40Hz BBT music therapy		
		VS/UWS	MCS	All	VS/UWS	MCS	All
		(n=10)	(n=9)	(n=19)	(n=10)	(n=11)	(n=21)
**EEG**							
Prefrontal pole	t1	0.653±0.215	0.793±0.111	0.720±0.184	0.664±0.090	0.676±0.095	0.670±0.090
	t2	0.777±0.295	0.839±0.188	0.807±0.245	0.752±0.173	0.819±0.221	0.787±0.198
	P-value (Cohen's *d*)	0.060 (0.698)	0.442 (0.270)	**0.043 (0.500)**	0.103 (0.088)	**0.042 (0.703)**	**0.007 (0.652)**
Frontal	t1	0.636±0.074	0.803±0.087	0.715±0.116	0.742±0.145	0.792±0.157	0.768±0.149
	t2	0.715±0.209	0.910±0.191	0.807±0.219	0.767±0.180	0.855±0.192	0.813±0.187
	P-value (Cohen's *d*)	0.209 (0.428)	0.148 (0.534)	**0.046 (0.493)**	0.634 (0.025)	0.357 (0.291)	0.287 (0.239)
Central	t1	0.601±0.141	0.838±0.131	0.713±0.180	0.691±0.108	0.811±0.128	0.754±0.132
	t2	0.748±0.220	0.894±0.122	0.817±0.191	0.794±0.209	0.859±0.164	0.828±0.185
	P-value (Cohen's *d*)	**0.031 (0.808)**	0.295 (0.373)	**0.016 (0.613)**	0.122 (0.103)	0.486 (0.218)	0.109 (0.366)
Parietal	t1	0.616±0.071	0.784±0.091	0.695±0.117	0.660±0.130	0.799±0.173	0.733±0.167
	t2	0.712±0.160	0.862±0.112	0.783±0.156	0.799±0.297	0.863±0.150	0.832±0.228
	P-value (Cohen's *d*)	0.074 (0.639)	**0.029 (0.884)**	**0.006 (0.716)**	0.119 (0.139)	0.332 (0.307)	0.060 (0.435)
Occipital	t1	0.642±0.152	0.827±0.101	0.730±0.158	0.732±0.240	0.780±0.179	0.757±0.206
	t2	0.687±0.142	0.814±0.104	0.747±0.139	0.842±0.331	0.848±0.177	0.845±0.255
	P-value (Cohen's *d*)	0.274 (0.391)	0.778 (0.097)	0.544 (0.142)	0.236 (0.110)	0.416 (0.256)	0.142 (0.333)
Temporal	t1	0.632±0.093	0.784±0.081	0.704±0.115	0.767±0.141	0.802±0.141	0.785±0.139
	t2	0.788±0.222	0.918±0.163	0.850±0.202	0.802±0.217	0.886±0.169	0.846±0.193
	P-value (Cohen's *d*)	**0.030 (0.816)**	0**.007 (1.207)**	**<0.001 (0.944)**	0.535 (0.035)	0.180 (0.434)	0.138 (0.337)
Central-prefrontal pole	t1	0.837±0.132	0.978±0.104	0.904±0.137	0.844±0.099	0.890±0.092	0.868±0.096
	t2	0.920±0.230	0.996±0.140	0.956±0.192	0.934±0.170	0.987±0.169	0.962±0.168
	P-value (Cohen's *d*)	0.171 (0.470)	0.781 (0.096)	0.217 (0.294)	0.087 (0.090)	0.124 (0.506)	**0.019 (0.556)**
Central-frontal	t1	0.799±0.063	0.979±0.123	0.885±0.131	0.859±0.121	0.920±0.123	0.891±0.123
	t2	0.860±0.211	1.019±0.109	0.935±0.185	0.908±0.155	1.000±0.186	0.956±0.174

	P-value (Cohen's *d*)	0.342 (0.318)	0.511 (0.229)	0.231 (0.284)	0.350 (0.051)	0.260 (0.360)	0.131 (0.344)
Central-parietal	t1	0.818±0.085	0.978±0.138	0.894±0.137	0.838±0.122	0.937±0.137	0.890±0.137
	t2	0.858±0.175	1.000±0.102	0.925±0.159	0.941±0.231	0.989±0.152	0.966±0.191
	P-value (Cohen's *d*)	0.464 (0.242)	0.669 (0.229)	0.385 (0.204)	0.136 (0.103)	0.449 (0.238)	0.105 (0.371)
Central-occipital	t1	0.831±0.107	0.994±0.098	0.908±0.130	0.888±0.155	0.917±0.130	0.903±0.139
	t2	0.877±0.169	0.987±0.086	0.929±0.144	0.982±0.262	0.982±0.155	0.982±0.207
	P-value (Cohen's *d*)	0.184 (0.332)	0.979 (0.050)	0.505 (0.156)	0.274 (0.094)	0.284 (0.307)	0.086 (0.395)
Central-anterior temporal	t1	0.802±0.093	0.960±0.078	0.877±0.117	0.852±0.144	0.909±0.121	0.882±0.132
	t2	0.870±0.188	0.992±0.095	0.928±0.160	0.964±0.250	1.011±0.192	0.988±0.217
	P-value (Cohen's *d*)	0.152 (0.495)	0.405 (0.293)	0.087 (0.415)	0.130 (0.112)	0.178 (0.437)	**0.036 (0.489)**
Central-middle temporal	t1	0.764±0.106	0.983±0.168	0.867±0.175	0.898±0.197	0.927±0.130	0.913±0.162
	t2	0.856±0.216	0.976±0.083	0.912±0.174	0.900±0.205	0.991±0.176	0.948±0.191
	P-value (Cohen's *d*)	0.072 (0.644)	0.906 (0.041)	0.247 (0.275)	0.977 (0.002)	0.400 (0.265)	0.492 (0.153)
**fNIRS**							
Average FC of HbO	t1	0.329±0.213	0.341±0.173	0.334±0.190	0.324±0.131	0.387±0.160	0.357±0.147
	t2	0.339±0.221	0.459±0.300	0.396±0.261	0.434±0.233	0.559±0.204	0.499±0.222
	P-value (Cohen's *d*)	0.814 (0.766)	0.209 (0.455)	0.211 (0.298)	0.082 (0.110)	**0.004 (1.106)**	**<0.001 (0.863)**

Abbreviations: BBT: Binaural Beat Therapy, HbO: oxygenated hemoglobin, VS/UWS: vegetative state/unresponsive wakefulness syndrome, MCS: minimally conscious state, EEG: electroencephalogram, fNIRS: functional near-infrared spectroscopy. Bold text indicates statistically significant differences between post-therapy (t2) and baseline (t1) time points: **P<0.05.**

Supplementary Table 4. Between-Group Comparisons of ΔEEG and fNIRS Biomarkers in DOC Subgroups

	Control Group (Δ=t2-t1)			40Hz-BBT music therapy (Δ=t2-t1)			P_1 value (Cohen's *d*)	P_2 value (Cohen's *d*)	P_3 value (Cohen's *d*)
	VS/UWS	MCS	All	VS/UWS	MCS	All			
	(n=10)	(n=9)	(n=19)	(n=10)	(n=11)	(n=21)			
**EEG**									
Prefrontal pole	0.124±0.178	0.051±0.169	0.090±0.173	0.088±0.153	0.143±0.204	0.117±0.179	0.791	0.691	0.415
Frontal	0.079±0.185	0.107±0.201	0.092±0.188	0.025±0.160	0.063±0.217	0.045±0.188	0.896	0.581	0.704
Central	0.147±0.182	0.056±0.149	0.104±0.169	0.103±0.191	0.047±0.217	0.074±0.202	0.386	0.888	0.268
Parietal	0.097±0.151	0.078±0.088	0.088±0.122	0.139±0.254	0.064±0.207	0.099±0.228	**0.012**	0.082	0.126
Occipital	0.045±0.114	-0.013±0.131	0.017±0.122	0.110±0.274	0.068±0.267	0.099±0.228	**0.016**	**0.015**	0.244
Temporal	0.156±0.191	0.134±0.111	0.146±0.154	0.035±0.170	0.085±0.195	0.061±0.180	0.568	0.841	0.215
Central-prefrontal pole	0.083±0.176	0.017±0.179	0.052±0.176	0.090±0.148	0.098±0.193	0.094±0.169	0.852	0.656	0.489
Central-frontal	0.060±0.189	0.040±0.175	0.051±0.178	0.050±0.159	0.080±0.222	0.094±0.169	0.642	0.757	0.294
[illegible]	[illegible]	[illegible]	[illegible]	[illegible]	[illegible]	[illegible]			
Central-occipital	0.046±0.138	-0.007±0.133	0.021±0.135	0.094±0.196	0.065±0.212	0.079±0.200	0.178	0.265	**0.007**
Central-anterior temporal	0.069±0.138	0.032±0.110	0.051±0.124	0.112±0.212	0.102±0.234	0.107±0.218	**0.006**	0.338	0.245
Central-middle temporal	0.092±0.143	-0.007±0.178	0.045±0.164	0.002±0.215	0.064±0.242	0.035±0.226	0.187	0.678	0.194
**fNIRS**									
Average FC of HbO	0.011±0.140	0.118±0.259	0.061±0.206	0.110±0.177	0.172±0.155	0.142±0.165	0.282	0.647	0.047

Δt2-t1 represents the pre-to-post treatment change score. All p-values were derived from two-tailed independent-samples Student's t-tests comparing scores between the control group and the 40 Hz BBT music therapy group.
P_1: Overall between-group comparison (all control participants, n=19 vs. all 40 Hz BBT participants, n=21);
P_2: VS/UWS subgroup comparison (control VS/UWS, n=10 vs. 40 Hz BBT VS/UWS, n=10);
P_3: MCS subgroup comparison (control MCS, n=9 vs. 40 Hz BBT MCS, n=11).
Cohen's *d* values represent the effect size for the corresponding comparisons. Bold text indicates statistically significant differences ($P<0.05$).
Abbreviations: EEG, electroencephalography; fNIRS, functional near-infrared spectroscopy; FC, functional connectivity; HbO, oxygenated hemoglobin; VS/UWS, vegetative state/unresponsive wakefulness syndrome; MCS, minimally conscious state.
