# Supplementary material for: Cortical processing modulation in DOC by preferred music-coupled 40 Hz binaural stimulation: an exploratory EEG-fNIRS study
Source: Front Psychol. 2026 Jul 13;17:1783416. doi: 10.3389/fpsyg.2026.1783416 (PMC13402131; doi:10.3389/fpsyg.2026.1783416)
Supplement: Supplementary file 3 [file Table_1.DOCX]

**Supplementary Table 2**. Pre- and Post-treatment Comparison of CRS-R Scores

|  | Control group | | | 40 Hz BBT music therapy | | |  |
| --- | --- | --- | --- | --- | --- | --- | --- |
|  | Time points | Mean±SD | Δ (t2-t1) | Time points | Mean±SD | Δ (t2-t1) | P_2_ value (cohens’*d*) |
| CRS-R scores | t1 | 5.00±2.71 | 1.89±2.28 | t1 | 5.29±2.81 | 2.86±3.72 |  |
|  | t2 | 6.89±4.46 |  | t2 | 8.14±4.82 |  |  |
|  | P_1_ value (cohens’*d*) | **<0.01 (0.83)** |  | P value (cohens’*d*) | **<0.01 (0.77)** |  | 0.33 (0.31) |
| Auditory | t1 | 0.95±0.62 | 0.42±0.77 | t1 | 1.19±0.51 | 0.52±0.87 |  |
|  | t2 | 1.37±0.83 |  | t2 | 1.71±0.78 |  |  |
|  | P_1_ value (cohens’*d*) | **0.03 (0.55)** |  | P value (cohens’*d*) | **0.01 (0.60)** |  | 0.70 (0.12) |
| Visual perception | t1 | 1.05±1.08 | 0.53±0.77 | t1 | 1.05±1.07 | 0.76±1.22 |  |
|  | t2 | 1.58±1.43 |  | t2 | 1.81±1.40 |  |  |
|  | P_1_ value (cohens’*d*) | **0.01 (0.68)** |  | P value (cohens’*d*) | **0.01 (0.62)** |  | 0.48 (0.22) |
| Motor | t1 | 1.42±0.61 | 0.53±0.77 | t1 | 1.57±0.75 | 0.71±1.06 |  |
|  | t2 | 1.95±1.13 |  | t2 | 2.29±1.38 |  |  |
|  | P_1_ value (cohens’*d*) | **0.01 (0.68)** |  | P value (cohens’*d*) | **0.01 (0.68)** |  | 0.53 (0.19) |
| Expressive speech | t1 | 0.37±0.68 | 0.05±0.23 | t1 | 0.33±0.58 | 0.10±0.30 |  |
|  | t2 | 0.42±0.69 |  | t2 | 0.43±0.68 |  |  |
|  | P_1_ value (cohens’*d*) | 0.33 (0.23) |  | P value (cohens’*d*) | 0.16 (0.32) |  | 0.62 (0.18) |
| Communication | t1 | 0.05±0.23 | 0.11±0.32 | t1 | 0.10±0.30 | 0.14±0.48 |  |
|  | t2 | 0.16±0.37 |  | t2 | 0.24±0.54 |  |  |
|  | P_1_ value (cohens’*d*) | 0.16 (0.33) |  | P value (cohens’*d*) | 0.19 (0.30) |  | 0.77 (0.07) |
| Arousal | t1 | 1.16±0.37 | 0.26±0.45 | t1 | 1.05±0.74 | 0.62±0.67 |  |
|  | t2 | 1.42±0.61 |  | t2 | 1.67±1.06 |  |  |
|  | P_1_ value (cohens’*d*) | **0.02 (0.58)** |  | P value (cohens’*d*) | **<0.01 (0.93)** |  | 0.06 (0.62) |

P_1_ values were derived from paired t-tests, comparing pre-treatment (t1) and post-treatment (t2) scores within each group.

P_2_ values were derived from two-tailed independent-samples Student’s t-tests, comparing the change scores (Δ= t2-t1) between the two groups.

Cohen’s *d* values represent the effect size for the corresponding comparisons.

**Abbreviations:** CRS-R: Coma Recovery Scale-Revised; Bold text indicates statistically significant differences between post-therapy (t2) and baseline (t1) measurements: **P<0.05** (two-tailed paired Student's t-tests for the six CRS-R subscales).

**Supplementary Table 3**. Pre- and Post-treatment Comparison of EEG and fNIRS Biomarkers

|  | | Control group | | | 40Hz BBT music therapy | | |
| --- | --- | --- | --- | --- | --- | --- | --- |
|  | | VS/UWS | MCS | All | VS/UWS | MCS | All |
|  | | (n=10) | (n=9) | (n=19) | (n=10) | (n=11) | (n=21) |
| **EEG** | |  |  |  |  |  |  |
| Prefrontal pole | t1 | 0.653±0.215 | 0.793±0.111 | 0.720±0.184 | 0.664±0.090 | 0.676±0.095 | 0.670±0.090 |
|  | t2 | 0.777±0.295 | 0.839±0.188 | 0.807±0.245 | 0.752±0.173 | 0.819±0.221 | 0.787±0.198 |
|  | P-value (Cohen's *d*) | 0.060 (0.698) | 0.442 (0.270) | **0.043 (0.500)** | 0.103 (0.088) | **0.042 (0.703)** | **0.007 (0.652)** |
| Frontal | t1 | 0.636±0.074 | 0.803±0.087 | 0.715±0.116 | 0.742±0.145 | 0.792±0.157 | 0.768±0.149 |
|  | t2 | 0.715±0.209 | 0.910±0.191 | 0.807±0.219 | 0.767±0.180 | 0.855±0.192 | 0.813±0.187 |
|  | P-value (Cohen's *d*) | 0.209 (0.428) | 0.148 (0.534) | **0.046 (0.493)** | 0.634 (0.025) | 0.357 (0.291) | 0.287 (0.239) |
| Central | t1 | 0.601±0.141 | 0.838±0.131 | 0.713±0.180 | 0.691±0.108 | 0.811±0.128 | 0.754±0.132 |
|  | t2 | 0.748±0.220 | 0.894±0.122 | 0.817±0.191 | 0.794±0.209 | 0.859±0.164 | 0.828±0.185 |
|  | P-value (Cohen's *d*) | **0.031 (0.808)** | 0.295 (0.373) | **0.016 (0.613)** | 0.122 (0.103) | 0.486 (0.218) | 0.109 (0.366) |
| Parietal | t1 | 0.616±0.071 | 0.784±0.091 | 0.695±0.117 | 0.660±0.130 | 0.799±0.173 | 0.733±0.167 |
|  | t2 | 0.712±0.160 | 0.862±0.112 | 0.783±0.156 | 0.799±0.297 | 0.863±0.150 | 0.832±0.228 |
|  | P-value (Cohen's *d*) | 0.074 (0.639) | **0.029 (0.884)** | **0.006 (0.716)** | 0.119 (0.139) | 0.332 (0.307) | 0.060 (0.435) |
| Occipital | t1 | 0.642±0.152 | 0.827±0.101 | 0.730±0.158 | 0.732±0.240 | 0.780±0.179 | 0.757±0.206 |
|  | t2 | 0.687±0.142 | 0.814±0.104 | 0.747±0.139 | 0.842±0.331 | 0.848±0.177 | 0.845±0.255 |
|  | P-value (Cohen's *d*) | 0.274 (0.391) | 0.778 (0.097) | 0.544 (0.142) | 0.236 (0.110) | 0.416 (0.256) | 0.142 (0.333) |
| Temporal | t1 | 0.632±0.093 | 0.784±0.081 | 0.704±0.115 | 0.767±0.141 | 0.802±0.141 | 0.785±0.139 |
|  | t2 | 0.788±0.222 | 0.918±0.163 | 0.850±0.202 | 0.802±0.217 | 0.886±0.169 | 0.846±0.193 |
|  | P-value (Cohen's *d*) | **0.030 (0.816)** | 0**.007 (1.207)** | **<0.001 (0.944)** | 0.535 (0.035) | 0.180 (0.434) | 0.138 (0.337) |
| Central-prefrontal pole | t1 | 0.837±0.132 | 0.978±0.104 | 0.904±0.137 | 0.844±0.099 | 0.890±0.092 | 0.868±0.096 |
|  | t2 | 0.920±0.230 | 0.996±0.140 | 0.956±0.192 | 0.934±0.170 | 0.987±0.169 | 0.962±0.168 |
|  | P-value (Cohen's *d*) | 0.171 (0.470) | 0.781 (0.096) | 0.217 (0.294) | 0.087 (0.090) | 0.124 (0.506) | **0.019 (0.556)** |
| Central-frontal | t1 | 0.799±0.063 | 0.979±0.123 | 0.885±0.131 | 0.859±0.121 | 0.920±0.123 | 0.891±0.123 |
|  | t2 | 0.860±0.211 | 1.019±0.109 | 0.935±0.185 | 0.908±0.155 | 1.000±0.186 | 0.956±0.174 |
|  | P-value (Cohen's *d*) | 0.342 (0.318) | 0.511 (0.229) | 0.231 (0.284) | 0.350 (0.051) | 0.260 (0.360) | 0.131 (0.344) |
| Central-parietal | t1 | 0.818±0.085 | 0.978±0.138 | 0.894±0.137 | 0.838±0.122 | 0.937±0.137 | 0.890±0.137 |
|  | t2 | 0.858±0.175 | 1.000±0.102 | 0.925±0.159 | 0.941±0.231 | 0.989±0.152 | 0.966±0.191 |
|  | P-value (Cohen's *d*) | 0.464 (0.242) | 0.669 (0.229) | 0.385 (0.204) | 0.136 (0.103) | 0.449 (0.238) | 0.105 (0.371) |
| Central-occipital | t1 | 0.831±0.107 | 0.994±0.098 | 0.908±0.130 | 0.888±0.155 | 0.917±0.130 | 0.903±0.139 |
|  | t2 | 0.877±0.169 | 0.987±0.086 | 0.929±0.144 | 0.982±0.262 | 0.982±0.155 | 0.982±0.207 |
|  | P-value (Cohen's *d*) | 0.184 (0.332) | 0.979 (0.050) | 0.505 (0.156) | 0.274 (0.094) | 0.284 (0.307) | 0.086 (0.395) |
| Central-anterior temporal | t1 | 0.802±0.093 | 0.960±0.078 | 0.877±0.117 | 0.852±0.144 | 0.909±0.121 | 0.882±0.132 |
|  | t2 | 0.870±0.188 | 0.992±0.095 | 0.928±0.160 | 0.964±0.250 | 1.011±0.192 | 0.988±0.217 |
|  | P-value (Cohen's *d*) | 0.152 (0.495) | 0.405 (0.293) | 0.087 (0.415) | 0.130 (0.112) | 0.178 (0.437) | **0.036 (0.489)** |
| Central-middle temporal | t1 | 0.764±0.106 | 0.983±0.168 | 0.867±0.175 | 0.898±0.197 | 0.927±0.130 | 0.913±0.162 |
|  | t2 | 0.856±0.216 | 0.976±0.083 | 0.912±0.174 | 0.900±0.205 | 0.991±0.176 | 0.948±0.191 |
|  | P-value (Cohen's *d*) | 0.072 (0.644) | 0.906 (0.041) | 0.247 (0.275) | 0.977 (0.002) | 0.400 (0.265) | 0.492 (0.153) |
| **fNIRS** |  |  |  |  |  |  |  |
| Average FC of HbO | t1 | 0.329±0.213 | 0.341±0.173 | 0.334±0.190 | 0.324±0.131 | 0.387±0.160 | 0.357±0.147 |
|  | t2 | 0.339±0.221 | 0.459±0.300 | 0.396±0.261 | 0.434±0.233 | 0.559±0.204 | 0.499±0.222 |
|  | P-value (Cohen's *d*) | 0.814 (0.766) | 0.209 (0.455) | 0.211 (0.298) | 0.082 (0.110) | **0.004 (1.106)** | **<0.001 (0.863)** |

**Abbreviations:** BBT: Binaural Beat Therapy, HbO: oxygenated hemoglobin, VS/UWS: vegetative state/unresponsive wakefulness syndrome, MCS: minimally conscious state, EEG: electroencephalogram, fNIRS: functional near-infrared spectroscopy. Bold text indicates statistically significant differences between post-therapy (t2) and baseline (t1) time points: **P<0.05.**

**Supplementary Table 4.** Between-Group Comparisons of ΔEEG and fNIRS Biomarkers in DOC Subgroups

|  | \| Control Group  (Δ=t2-t1) \| \| --- \| | | | 40Hz-BBT music therapy  (Δ=t2-t1) | | | P_1_ value  (Cohen's *d*) | P_2_ value  (Cohen's *d*) | P_3_ value  (Cohen's *d*) |
| --- | --- | --- | --- | --- | --- | --- | --- | --- | --- | --- |
|  | VS/UWS | MCS | AII | VS/UWS | MCS | AII |  |  |  |
|  | (n=10) | (n=9) | (n=19) | (n=10) | (n=11) | (n=21) |  |  |  |
| **EEG** |  |  |  |  |  |  |  |  |  |
| Prefrontal pole | 0.124±0.178 | 0.051±0.169 | 0.090±0.173 | 0.088±0.153 | 0.143±0.204 | 0.117±0.179 | 0.791 | 0.691 | 0.415 |
| Frontal | 0.079±0.185 | 0.107±0.201 | 0.092±0.188 | 0.025±0.160 | 0.063±0.217 | 0.045±0.188 | 0.896 | 0.581 | 0.704 |
| Central | 0.147±0.182 | 0.056±0.149 | 0.104±0.169 | 0.103±0.191 | 0.047±0.217 | 0.074±0.202 | 0.386 | 0.888 | 0.268 |
| Parietal | 0.097±0.151 | 0.078±0.088 | 0.088±0.122 | 0.139±0.254 | 0.064±0.207 | 0.099±0.228 | **0.012** | 0.082 | 0.126 |
| Occipital | 0.045±0.114 | -0.013±0.131 | 0.017±0.122 | 0.110±0.274 | 0.068±0.267 | 0.099±0.228 | **0.016** | **0.015** | 0.244 |
| Temporal | 0.156±0.191 | 0.134±0.111 | 0.146±0.154 | 0.035±0.170 | 0.085±0.195 | 0.061±0.180 | 0.568 | 0.841 | 0.215 |
| Central-prefrontal pole | 0.083±0.176 | 0.017±0.179 | 0.052±0.176 | 0.090±0.148 | 0.098±0.193 | 0.094±0.169 | 0.852 | 0.656 | 0.489 |
| Central-frontal | 0.060±0.189 | 0.040±0.175 | 0.051±0.178 | 0.050±0.159 | 0.080±0.222 | 0.094±0.169 | 0.642 | 0.757 | 0.294 |
| Central-parietal | 0.040±0.163 | 0.022±0.150 | 0.031±0.153 | 0.103±0.199 | 0.052±0.218 | 0.076±0.206 | 0.071 | 0.455 | 0.156 |
| Central-occipital | 0.046±0.138 | -0.007±0.133 | 0.021±0.135 | 0.094±0.196 | 0.065±0.212 | 0.079±0.200 | 0.178 | 0.265 | **0.007** |
| Central-anterior temporal | 0.069±0.138 | 0.032±0.110 | 0.051±0.124 | 0.112±0.212 | 0.102±0.234 | 0.107±0.218 | **0.006** | 0.338 | 0.245 |
| Central-middle temporal | 0.092±0.143 | -0.007±0.178 | 0.045±0.164 | 0.002±0.215 | 0.064±0.242 | 0.035±0.226 | 0.187 | 0.678 | 0.194 |
| **fNIRS** |  |  |  |  |  |  |  |  |  |
| Average FC of HbO | 0.011±0.140 | 0.118±0.259 | 0.061±0.206 | 0.110±0.177 | 0.172±0.155 | 0.142±0.165 | 0.282 | 0.647 | 0.047 |

Δt2-t1 represents the pre-to-post treatment change score. All p-values were derived from two-tailed independent-samples Student’s t-tests comparing scores between the control group and the 40 Hz BBT music therapy group.

P_1_: Overall between-group comparison (all control participants, n=19 vs. all 40 Hz BBT participants, n=21);

P_2_: VS/UWS subgroup comparison (control VS/UWS, n=10 vs. 40 Hz BBT VS/UWS, n=10);

P_3_: MCS subgroup comparison (control MCS, n=9 vs. 40 Hz BBT MCS, n=11).

Cohen’s *d* values represent the effect size for the corresponding comparisons. Bold text indicates statistically significant differences (P<0.05).

**Abbreviations:** EEG, electroencephalography; fNIRS, functional near-infrared spectroscopy; FC, functional connectivity; HbO, oxygenated hemoglobin; VS/UWS, vegetative state/unresponsive wakefulness syndrome; MCS, minimally conscious state.
