# Supplementary material for: Cortical processing modulation in DOC by preferred music-coupled 40 Hz binaural stimulation: an exploratory EEG-fNIRS study
Source: Front Psychol. 2026 Jul 13;17:1783416. doi: 10.3389/fpsyg.2026.1783416 (PMC13402131; doi:10.3389/fpsyg.2026.1783416)
Supplement: Supplementary file 4 [file Table_2.DOCX]

**Supplementary Table 1**.fNIRS Channel Assignment to Anatomical ROIs via MNI Coordinates

| **Channel ID** | **MNI Coordinates (mm)** | | | **Brodmann Area** | **ROI** |
| --- | --- | --- | --- | --- | --- |
|  | **X** | **Y** | **Z** |  |  |
| CH1 (S1-D1) | 62 | -7 | 42 | BA 6 | RMC |
| CH2 (S1-D12) | 54 | -7 | 54 | BA 6 | RMC |
| CH3 (S2-D2) | 55 | 41 | -8 | BA 47 | RIFG |
| CH4 (S2-D7) | 61 | 26 | 10 | BA 47 | RIFG |
| CH5 (S3-D2) | 41 | 63 | -7 | BA 10 | RPFC |
| CH6 (S3-D3) | 14 | 72 | -8 | BA 10 | RPFC |
| CH7 (S3-D8) | 28 | 68 | 11 | BA 10 | RPFC |
| CH8 (S4-D3) | -12 | 72 | -8 | BA 10 | LPFC |
| CH9 (S4-D4) | -38 | 63 | -13 | BA 10 | LPFC |
| CH10 (S4-D9) | -26 | 70 | 10 | BA 10 | LPFC |
| CH11 (S5-D4) | -53 | 42 | -15 | BA 47 | LIFG |
| CH12 (S5-D10) | -59 | 26 | -1 | BA 47 | LIFG |
| CH13 (S6-D11) | -45 | -5 | 62 | BA 6 | LMC |
| CH14 (S6-D15) | -32 | -6 | 69 | BA 6 | LMC |
| CH15 (S7-D12) | 45 | -27 | 68 | BA 3 | RPSC |
| CH16 (S7-D13) | 34 | -27 | 73 | BA 4 | RMC |
| CH17 (S8-D2) | 49 | 51 | 9 | BA 46 | RDLPFC |
| CH18 (S8-D7) | 53 | 35 | 26 | BA 46 | RDLPFC |
| CH19 (S8-D8) | 37 | 54 | 26 | BA 10 | RPFC |
| CH21 (S9-D8) | 15 | 65 | 28 | BA 10 | RPFC |
| CH22 (S9-D9) | -13 | 66 | 28 | BA 10 | LPFC |
| CH23 (S10-D4) | -49 | 52 | 2 | BA 46 | LDLPFC |
| CH24 (S10-D9) | -37 | 58 | 24 | BA 10 | LPFC |
| CH25 (S10-D10) | -54 | 37 | 18 | BA 46 | LDLPFC |
| CH26 (S11-D5) | -66 | -6 | 35 | BA 6 | LMC |
| CH27 (S11-D11) | -56 | -7 | 53 | BA 6 | LMC |
| CH28 (S12-D12) | 44 | -5 | 63 | BA 6 | RMC |
| CH29 (S12-D13) | 32 | -6 | 69 | BA 6 | RMC |
| CH30 (S13-D11) | -47 | -29 | 67 | BA 3 | LPSC |
| CH31 (S13-D15) | -35 | -28 | 73 | BA 4 | LMC |
| CH32 (S14-D5) | -69 | -31 | 38 | BA 2 | LPSC |
| CH33 (S14-D11) | -58 | -28 | 54 | BA 2 | LPSC |
| CH34 (S15-D1) | 66 | -30 | 47 | BA 2 | RPSC |
| CH35 (S15-D12) | 55 | -28 | 58 | BA 2 | RPSC |
| CH36 (S16-D6) | 35 | -92 | 26 | BA 19 | ROL |
| CH37 (S17-D6) | 24 | -104 | 10 | BA 18 | ROL |
| CH38 (S17-D16) | 16 | -107 | -5 | BA 18 | ROL |
| CH39 (S18-D14) | -35 | -93 | 23 | BA 19 | LOL |
| CH40 (S19-D14) | -24 | -105 | 7 | BA 18 | LOL |
| CH41 (S19-D16) | -13 | -106 | -7 | BA 18 | LOL |
| CH42 (S20-D13) | 22 | -27 | 77 | BA 4 | RMC |
| CH43 (S21-D15) | -20 | -6 | 77 | BA 6 | LMC |
| CH44 (S22-D15) | -22 | -28 | 77 | BA 4 | LMC |
| CH45 (S23-D13) | 22 | -4 | 76 | BA 6 | RMC |
| CH46 (S24-D6) | 15 | -101 | 25 | BA 19 | ROL |
| CH47 (S24-D14) | -12 | -101 | 24 | BA 19 | LOL |

Abbreviations: BA, Brodmann Area; ROI, region of interest; LDLPFC, left dorsolateral prefrontal cortex; RDLPFC, right dorsolateral prefrontal cortex; LPFC, left prefrontal cortex; RPFC, right prefrontal cortex; LIFG, left inferior frontal gyrus; RIFG, right inferior frontal gyrus; LMC, left motor cortex; RMC, right motor cortex; LPSC, primary somatosensory cortex; RPSC, primary somatosensory cortex; LOL, left occipital lobe; ROL, right occipital lobe.
